# Investigating Aging‐Related Endometrial Dysfunction Using Endometrial Organoids

**DOI:** 10.1111/cpr.13780

**Published:** 2024-12-18

**Authors:** Minghui Lu, Yanli Han, Yu Zhang, Ruijie Yu, Yining Su, Xueyao Chen, Boyang Liu, Tao Li, Rusong Zhao, Han Zhao

**Affiliations:** ^1^ State Key Laboratory of Reproductive Medicine and Offspring Health, Center for Reproductive Medicine, Institute of Women, Children and Reproductive Health Shandong University Jinan China; ^2^ National Research Center for Assisted Reproductive Technology and Reproductive Genetics Shandong University Jinan China; ^3^ Key Laboratory of Reproductive Endocrinology (Shandong University) Ministry of Education Jinan China; ^4^ Shandong Technology Innovation Center for Reproductive Health Jinan China; ^5^ Shandong Provincial Clinical Research Center for Reproductive Health Jinan China; ^6^ Shandong Key Laboratory of Reproductive Research and Birth Defect Prevention Jinan Shandong China; ^7^ Research Unit of Gametogenesis and Health of ART‐Offspring Chinese Academy of Medical Sciences (No. 2021RU001) Jinan China; ^8^ Department of Obstetrics and Gynecology, Shandong Provincial Hospital Shandong First Medical University Jinan China; ^9^ The Affiliated Suzhou Hospital of Nanjing Medical University, Suzhou Municipal Hospital, Gusu School Nanjing Medical University Suzhou Jiangsu China

**Keywords:** endometrial ageing, fibrosis, inflammation, organoids, PI3K/AKT/FOXO1, receptivity

## Abstract

Ageing of the endometrium is a critical factor that affects reproductive health, yet its intricate mechanisms remain poorly explored. In this study, we performed transcriptome profiling and experimental verification of endometrium and endometrial organoids from young and advanced age females, to elucidate the underlying mechanisms and to explore novel treatment strategies for endometrial ageing. First, we found that age‐associated decline in endometrial functions including fibrosis and diminished receptivity, already exists in reproductive age. Subsequently, based on RNA‐seq analysis, we identified several changes in molecular processes affected by age, including fibrosis, imbalanced inflammatory status including Th1 bias in secretory phase, cellular senescence and abnormal signalling transduction in key pathways, with all processes been further validated by molecular experiments. Finally, we uncovered for the first time that PI3K‐AKT‐FOXO1 signalling pathway is overactivated in ageing endometrium and is closely correlated with fibrosis and impaired receptivity characteristics of ageing endometrium. Blocking or activation of PI3K by LY294002 or 740Y‐P could attenuate the effect of ageing or accelerate dysfunction of endometrial organoids. This discovery is expected to bring new breakthroughs for understanding the pathophysiological processes associated with endometrial ageing, as well as treatment strategies to improve reproductive outcomes in women of advanced reproductive age.

## Graphical abstract

Age‐associated fibrosis, imbalanced inflammatory homeostasis including Th1 bias in secretory phase, cellular senescence and decline of receptivity exist in human endometrium of advanced age. Overactivation of PI3K‐AKT‐FOXO1 signalling pathway could give rise to those characteristics above.

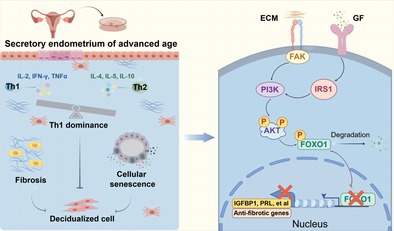

## 1 Introduction

Ageing is a complex process characterised by a gradual decline in physiological structure and cellular function [1, 2, 3]. As a pivotal functional system, reproductive system ageing due to advancing maternal age significantly compromises fertility and becomes more pronounced beyond 35 years of age [4, 5, 6, 7]. Traditionally, age‐related reproductive dysfunction has been attributed primarily to ovarian ageing and hormonal insufficiency [8, 9, 10, 11]. However, numerous studies have shown that ageing of the endometrium also influences the implantation rate, clinical pregnancy rate, and live birth rate in women of advanced age. This suggests ageing endometrium is equally important in age‐related reproductive decline [12, 13, 14], although some controversies persist [15, 16].

Numerous epidemiological evidence and experimental studies have supported a negative correlation between age and endometrial function. For instance, a retrospective study based on endometrial transcriptome datasets of 27 women aged 23–43 years revealed the significant changes in molecular processes occurring in the endometrium after 35 years of age. These changes include cell cycle arrest and up‐regulation of ciliary processes, which may contribute to diminished endometrial receptivity and reproductive success [17]. Moreover, using genome‐wide analysis, Rubel and Hewitt et al. have confirmed a decline in expression levels of oestrogen receptor alpha (ERα) and P4 receptor (PR) in the endometrium with advancing age, which may lead to compromised hormonal responsiveness [18, 19]. Emerging evidence has suggested that multiple molecular activities participate in endometrial ageing, including oxidative stress, inflammation, fibrosis, DNA damage response and cellular senescence, while the precise cellular and molecular signalling mechanisms remain poorly explored [20].

Given single cell line fail to replicate complicated cell communication and microenvironment, and animal models do not faithfully represent the dynamic human cycling endometrium, the need for a reliable preclinical study model becomes more pressing. We and others have recently demonstrated the derivation of human endometrial glandular organoids in vitro, effectively mirroring the characteristics of endometrial glands in vivo [21, 22, 23]. These endometrial organoids, derived from normal endometrium or endometrial pathologies such as endometrial cancer, endometriosis and endometrial hyperplasia, show long‐term expandability, transcriptomic stability, disease‐associated traits and gene mutations as well [23].

Here we constructed organoid models by endometrium samples ranging from young and advanced age females, with hormone induction to the proliferative phase and secretory phase. Applying transcriptome sequencing and functional experiments, we investigated changes in the function and molecular signalling changes associated with ageing phenotypes that occur in endometrium of advanced age compared to young age. Our data showed that age‐associated fibrosis, imbalanced inflammation, cellular senescence and decline of receptivity exist in both endometrium tissue and endometrial organoids of advanced age. In the following work, we identified the involvement of PI3K‐AKT‐FOXO1 signalling pathway in these findings, suggesting a significant role for the alternations in molecular processes of endometrial ageing. Additionally, our study has established an endometrial organoid biobank encompassing samples from both young and advanced age, thereby providing promising preclinical models for research and drug screening purposes.

## 2 Materials and Methods

### 2.1 Study Participants

The human endometrium and procedures involved in this study were approved by the Ethics Committee of Qilu Hospital of Shandong University (approval number: KYLL‐202204‐030) and the Ethics Committee of Shandong Provincial Hospital (approval number: SWYX:NO.2024‐204). Written informed consent was obtained from all human subjects, and the experiments were performed according to the principles of Declaration of Helsinki.

The human endometrium were collected at either proliferative or secretory phase of the menstrual cycle from women aged ≥ 35 years (advanced age group) and < 35 years (young group) during hysterectomy. All donors had regular menstrual cycles and had not received any hormonal treatment for at least 3 months prior to surgery. To explore the changes in differential gene expression profiles in the endometrium between young and advanced age females, we randomly selected 13 endometrial samples from the young group and 14 endometrial samples from the advanced age group to perform RNA sequencing, and all the samples were at the proliferative phase. The clinical information of all patients was listed in Table S1. Work with human endometrium was regulated by International Society for Stem Cell Research (ISSCR) ‘Guidelines for Stem Cell Research and Clinical Translation (2016)’ and Cell proliferation ‘General requirements for stem cells’ [24].

### 2.2 Construction, Hormone Treatment and PI3K‐AKT Activator/Inhibitor Treatment of Endometrial Organoids

The collected endometrium was placed in digestion solution containing 0.4 mg/mL collagenase V (Sigma, C‐9263), 1.25 U/mL dispase II (Sigma, D4693) and 10 μg/mL DNase I (Worthington, LS002139). After 20–30 min, the supernatant was slowly passed through a 40 μm cell strainer (Corning, 352340), and the glandular debris was centrifuged. The precipitate was then resuspended in DMEM/F12. After discarding the supernatant, DMEM/F12 and Matrigel (Corning, 356231) were added to the precipitate in a 1:3 ratio. A 40 μL drop of the mixture was added each well in a 24‐well plate and incubated at 37°C for 30 min. Each well was then covered with 500 μL organoid expansion medium (ExM), with the ExM being replaced every 3–4 days (see Table S2 for detailed composition).

According to the protocol of Fitzgerald et al. for induction of the proliferative and secretory phases [21], the organoids were treated with 10 nM estradiol (E2, Sigma, E2758) for 2 days to induce into the proliferative phase. Following this, the organoids were then treated with 10 nM E2, 1 μM medroxyprogesterone acetate (MPA, Selleck, S2567) and 1 μM cAMP (Sigma, D0627) for an additional 6 days to induce secretory phase (Details were shown in Table S2). As for PI3K‐AKT inhibitor LY294002 and PI3K‐AKT activator 740Y‐P and SC79 treatment, the organoids were treated with 20 μM LY294002 or 10 μM 740Y‐P or 10 μM SC79 for 4 days, respectively.

### 2.3 Quantitative Real‐Time PCR (qRT‐PCR)

Total RNA was isolated from organoids using RNA Extraction Reagent (Vazyme, R411). RNA (1 mg) was reverse transcribed into cDNA with HiScript III RT SuperMix for qPCR (Vazyme, R323). qRT‐PCR was performed with the Roche LightCycler480 Real‐Time PCR system using the TB Green Premix EX Taq (Takara, RR420). Differences among the expression levels of the target genes were estimated by the ΔΔCt (threshold cycling) method and normalised to the level of GAPDH. The primers used in this study were listed in Table S3.

### 2.4 Immunofluorescence Staining

Endometrium fixed in 4% paraformaldehyde (PFA) was dehydrated, embedded in paraffin, and sectioned at 5 μm. Organoids were fixed in 4% PFA, dehydrated, embedded in OCT and sectioned at 10 μm. For paraffin section, the slides were dewaxed in xylene and rehydrated through graded ethanol concentrations. For frozen section, the slides were fixed with 4% PFA and washed with PBS. Next, the slides were permeated with PBS containing 2% Triton X‐100 for 30 min at room temperature. Antigen retrieval was performed in citrate solution at 100°C for 20 min and then were blocked with QuickBlock Immunostaining Blocking Buffer (Beyotime, P0260) at room temperature for 1 h. After incubating with the primary antibodies (Table S4) overnight at 4°C, the slides were incubated with appropriate secondary antibody (Table S4) for 1 h, followed by staining with DAPI (Beyotime Biotechnology, C1002) at room temperature for 20 min. The slides were then mounted using Anti‐Fade Fluorescence Mounting Medium (Abcam, ab104135). Images were captured using the laser confocal microscope (Andor Dragonfly 200) and processed by Imaris x64 9.0.1.

### 2.5 Western Blot

The organoids were transferred to the digestion solution for shaking 20–30 min. The organoid fragments were collected and lysed following the instructions of the Minute Total Protein Extraction Kit (Invent Biotechnologies, SD‐001/SN‐002). After determination of the protein concentration, the total lysate was mixed with SDS loading buffer (Beyotime, P0015), and denatured at 100°C for 10 min before proceeding to SDS‐PAGE. Proteins were then transferred to polyvinylidene fluoride membranes via electroblotting. Blocked with QuickBlock Western Blocking Buffer (Beyotime, P0252) at room temperature for 20 min, and then incubated with primary antibodies (Table S4) overnight at 4°C. After that, the membranes were incubated with secondary antibodies (Table S4) for 1 h at room temperature. Finally, the membranes were imaged with a Bio‐Rad system. Band identification and quantification were performed using ImageLab software.

### 2.6 Masson Staining

Paraffin sections were prepared, dewaxed and hydrated. According to the instructions of Masson's Trichrome Staining Kit (Beyotime, C0189), the paraffin sections were stained in succession with haematoxylin staining, porceau‐acid fuchsin staining and light green staining. After dehydration, transparency and mounting, images were captured by an Aperio VERSA 8 (Leica Biosystems).

### 2.7 Inflammation Biomarker Preparations

The organoid culture medium was collected. Using the RayPlex Human Inflammation Array Kit 1 (RayBiotech, FAH‐INF‐1), the quantification of 13 cytokines/chemokine biomarkers associated with inflammation, including G‐CSF, IL‐13, IFNγ, IL‐2, TNFα, IL‐4, IL‐6, IL‐10, IL‐12p70, IL‐17A, IL‐1β, MCP‐1 and IL‐23p19 were performed by flow cytometric analysis following the instructions of the manufacturer. The values of PE MFI of all samples were acquired using BD FACSDiva Batch Analysis.

### 2.8 Bulk RNA‐Sequencing and Analysis

Total RNA of endometrium and organoids were extracted using RNA Extraction Reagent (Vazyme, R411). The RNA concentration was measured using Qubit RNA BR Assay Kits (Thermo Fisher, Q10211). The RNA integrity number (RIN) was assessed using the RNA 6000 Nano Kit (Agilent 5067‐1511) and the Agilent 2100 Bioanalyzer (Agilent Technologies, Palo Alto, CA, USA). RNA with the RIN ≥ 7 was used to construct the cDNA library using the VAHTS Universal V8 RNA‐seq Library Prep Kit for Illumina (Vazyme, NR605). The library quality was assessed using DNA 1000 Kit (Agilent 5067‐1504).

Transcriptome sequencing was performed on Illumina NovaSeq6000 by Tianjin Novogene Technology Co. Ltd. (Tianjin, China) and Illumina sequencing platform by LC‐BIO Co. Ltd. (HangZhou, China). For proliferative endometrium, genes with a false discovery rate (FDR) threshold < 0.05 and an absolute fold change > 2 were considered as DEGs; for the proliferative endometrial organoids, genes with a *p* value threshold < 0.05 and an absolute fold change > 2 were considered as DEGs; for the secretory endometrial organoids, genes with a FDR threshold < 0.05 and an absolute fold change > 2 were considered as DEGs (Table S5). Transcriptome analysis was performed using Omicshare, a real‐time interactive online data analysis platform (http://www.omicshare.com).

### 2.9 Statistical Analysis

Statistical analysis was performed using GraphPad Prism 8. Group comparisons employed unpaired *t*‐test or one‐way ANOVA. Only *p* < 0.05 was considered statistically significant. *p < 0.05, **p < 0.01, *** *p* < 0.001, ns, not significant. Staining slides were observed by two pathologists independently in a double‐blind fashion. Technical or biological replicates were described in each figure legend.

## 3 Results

### 3.1 Endometrial Function Is Declined in Advanced Age Women

**FIGURE 1:**
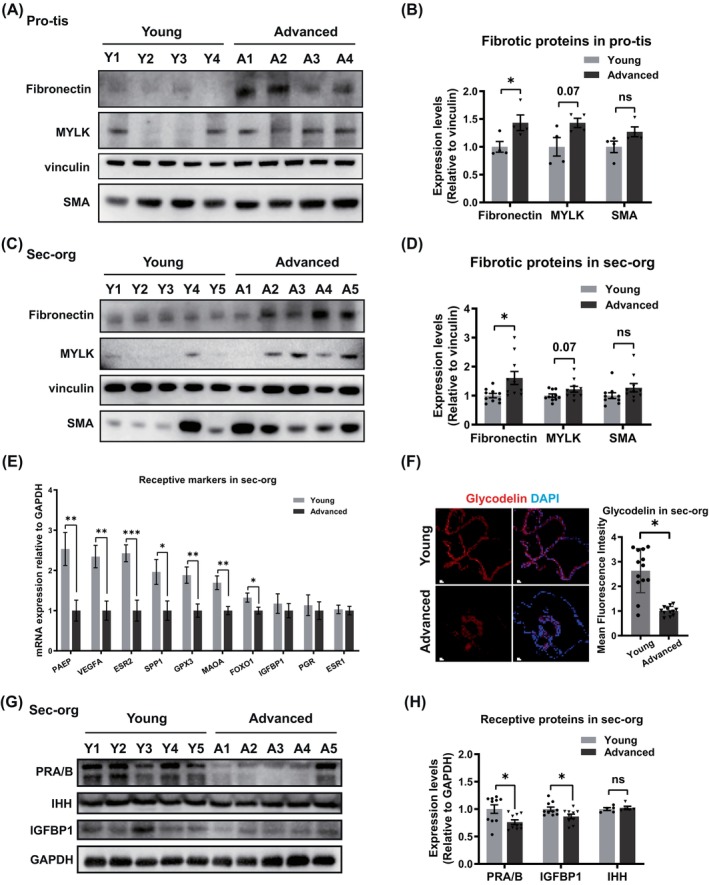
Increased fibrosis and declined endometrial receptivity in advanced age women. (A) Representative WB images of fibronectin, MYLK and SMA in pro‐tis from young and advanced age patients. (B) Relative levels of fibronectin, MYLK and SMA in pro‐tis from young and advanced age patients. (C) Representative WB images of fibronectin, MYLK and SMA in sec‐org derived from patients Y1–Y5 and A1–A5. (D) Relative levels of fibronectin, MYLK and SMA in sec‐org derived from young and advanced age patients. (E) The mRNA expression levels of receptive genes of sec‐org (*n* ≥ 8 biological replicates for each group). (F) Representative fluorescence images and mean fluorescence intensities for glycodelin in sec‐org. (*n* = 3 biological replicates for each group). (G) Representative WB images of PRA/B, IHH, IGFBP1 in sec‐org derived from patients Y1–Y5 and A1–A5. (H) Relative levels of PRA/B, IHH, IGFBP1 in sec‐org derived from young and advanced age patients. Scale bar = 50 μm. Data are presented as mean ± standard error of the mean (SEM) with two‐tailed Student's *t*‐test. *p < 0.05; **p < 0.01; ***p < 0.001.

Endometrium were collected from patients undergoing hysterectomy due to benign endometrial disease and benign cervical disease. We divided them into two groups based on age: < 35 years (young group) and ≥ 35 years (advanced age group) and analysed age‐associated differences of endometrial function. The mRNA expression of pro‐fibrotic genes (*MYLK, LAMA2, LAMC3, COL6A1, COL6A2, ITGA11*, etc.) and proteins levels of fibronectin and MYLK were up‐regulated in advanced age proliferative endometrial tissue (pro‐tis) (Figures 1A,B and S2A) [25, 26]. Masson staining further indicated an increased level of fibrosis in the advanced age endometrium, a phenotype that is closely related to impaired fertility (Figure S2B) [25].

We established organoid models using endometrium tissue from both young and advanced age individuals. We observed the remarkable resemblance in cellular composition and tissue architecture to the in vivo endometrium (Figure S1). The organoids were subjected with estradiol (E2) inducing to the proliferative phase (pro‐org), and followed by a combination of E2, medroxyprogesterone acetate (MPA) and *N*6,2′‐O‐dibutyryladenosine 3′,5′‐cyclic monophosphate sodium salt (cAMP) to induce to the secretory phase (sec‐org). Subsequently, the hormone‐stimulated organoids were collected for PCR assays, IF and western blot (WB) analysis as well. Our results revealed an increased fibrosis both in the pro‐org and sec‐org of advanced age group, which were consistent with advanced age endometrium (Figures 1C,D and S2C–H).

In addition, the protein levels of receptive marker such as PRA/B and IGFBP1 in secretory endometrium (sec‐tis) was significantly reduced in the advanced group (Figure S3A,B). Similarly, the mRNA expression of a variety of receptive genes and protein levels of PRA/B, IGFBP1 were significantly reduced in the advanced sec‐org, while there was no notable difference of IHH expression between two groups (Figures 1E–H and S3C–E). These results are similar to those reported by Berdiaki et al. [27]. Therefore, the above results suggested that age‐associated decline of endometrial function were already present in reproductive age, and the constructed endometrial organoids effectively capture age‐related function changes.

### 3.2 Advanced Age Endometrium Exhibited Fibrosis and Imbalanced Inflammatory Transcriptome Signature

**FIGURE 2:**
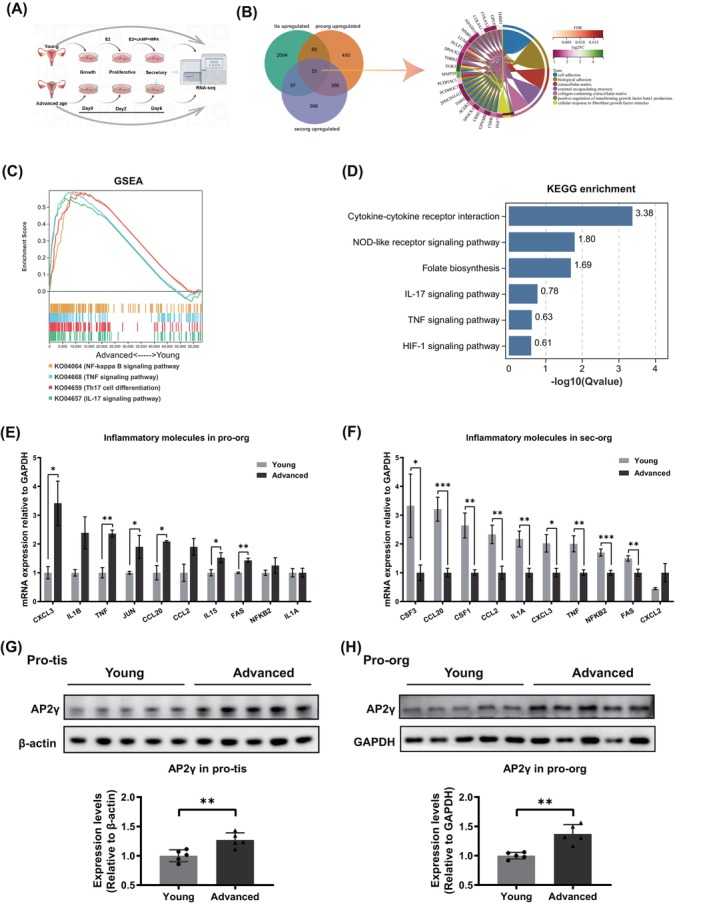
Advanced age endometrium exhibited a fibrosis transcriptome characterisation and menstrual cycle‐specific inflammatory transcriptome signature. (A) The workflow of organoid preparation and RNA‐seq analysis. Total mRNA samples from pro‐tis (young: *n* = 13, advanced: *n* = 14), pro‐org (young: *n* = 9, advanced: *n* = 7) and sec‐org (young: *n* = 13, advanced: *n* = 7) were sequenced. (B) Venn diagram displaying 53 DEGs screened from three groups (left). Gene Ontology (GO) circle plot showing the GO functions enriched by these 53 genes (right). (C) Gene set enrichment analysis (GSEA) of inflammation‐related pathways between young and advanced age pro‐tis. (D) KEGG enrichment analysis for genes that were down‐regulated in advanced age sec‐org. (E) The mRNA expression levels of inflammatory molecules in pro‐org (*n* ≥ 3 biological replicates for each group). (F) The mRNA expression levels of inflammatory molecules in sec‐org (*n* ≥ 5 biological replicates for each group). (G) Representative WB images and relative levels of AP2γ in pro‐tis from young and advanced age patients. (H) Representative WB images and relative levels of AP2γ in pro‐org derived from young and advanced age patients. Data are presented as mean ± standard error of the mean (SEM) with two‐tailed Student's *t*‐test in (E–H). Significance: *p < 0.05, **p < 0.01, ***p < 0.001.

To analyse the differences in gene expression between young and advanced age endometrium, total mRNA samples from pro‐tis, pro‐org and sec‐org were sequenced (Figure 2A). Based on gene expression profiles, the study samples clustered together with no clear outliers in both groups (Figure S4A). A total of 1174 significantly differentially expressed genes (DEGs), 1240 DEGs and 737 DEGs were identified in the endometrium, pro‐org and sec‐org, respectively (Table S5).

Fibrosis‐related pathways, such as focal adhesion, TGF‐beta signalling pathway and collagen fibril organisation, as well as response to growth factors were significantly up‐regulated in the advanced age pro‐tis and pro‐org (Figure S4B–G, Tables S7 and S8) [28, 29, 30, 31, 32, 33, 34, 35]. Furthermore, top up‐regulated GO terms in advanced pro‐org included ciliary plasm, axoneme and axonemal microtubule, which were consistent with previous studies on endometrial ageing, such as that by Devesa‐Peiro et al., in which most of affected functions were related to up‐regulation of ciliary process (Figure S4H) [17]. Based on a |log2(FoldChange)| > 0.8 and a *p* value < 0.05, 122 common up‐regulated genes were revealed between endometrium and pro‐org, and 419 common up‐regulated genes were found between pro‐org and sec‐org in the advanced age group. Functional enrichment analysis of these common genes strongly suggested fibrosis‐related patterns, partly reflects the transcriptional similarity between the organoids and the in vivo endometrium (Figure S5A–C, Table S6). Additionally, 53 up‐regulated common genes among advanced age pro‐tis, pro‐org and sec‐org showed significantly enriched GO terms, including cell adhesion and extracellular matrix (Figure 2B, Table S6). These results demonstrated advanced age endometrium possesses phenotypic and transcriptomic characteristics of fibrosis independent of menstrual cycle.

Fibrosis, as a pathological outcome of most chronic inflammatory diseases, is primarily regulated by senescence‐associated secretory phenotype (SASP), including pro‐inflammatory cytokines and metalloproteinases [36, 37, 38]. Next, we focused on changes of inflammatory status of ageing endometrium. We found a significant up‐regulation of pro‐inflammatory pathways such as NF‐kappa B signalling pathway, TNF signalling pathway, Th17 cell differentiation and IL17 signalling pathway in both advanced age pro‐tis and pro‐org (Figures 2C and S6A, Tables S7 and S8). However, pro‐inflammatory pathways were down‐regulated in the advanced age sec‐org (Figure 2D, Table S9). We speculate that this change may be due to the menstrual cycles, which was validated by qRT‐PCR, WB and multiplex cytokine detection with flow cytometry (Figures 2E–H and S6B–G). Besides, a large body of evidence has indicated that successful implantation is characterised by Th2 dominance, which mainly produce IL‐4, IL‐5 and IL‐10 [39, 40, 41]. In contrast, recurrent implantation failure (RIF) patients exhibit a Th1 bias, with increased production of IL‐2, IFNγ and TNF [41, 42, 43, 44, 45, 46]. Our findings showed that there was an increase in the production of IL2 and IFNγ, while IL10 secretion decreased in advanced age sec‐org, which suggests that the Th1/Th2 ratio is skewed towards the Th1 type. Therefore, these results validated the hypothesis proposed by our sequencing profile: dysregulation of inflammatory balance in ageing endometrium may potentially impact endometrial receptivity and embryo implantation.

### 3.3 Identification of Aging Transcriptomic Characteristics in Advanced Age Endometrium

**FIGURE 3:**
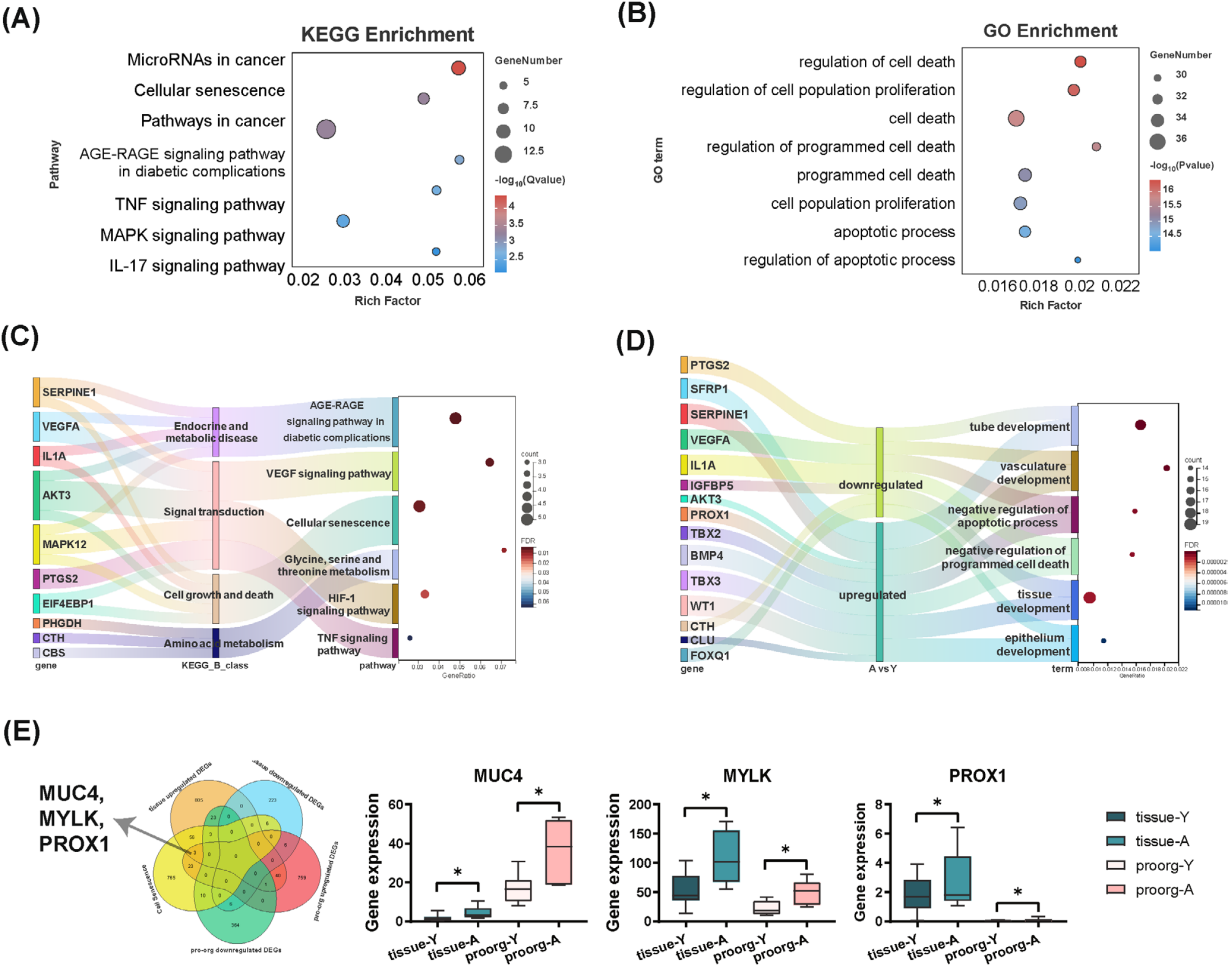
Identification of ageing transcriptome characterisation in advanced age endometrium. (A) KEGG enrichment analysis of SRGs of pro‐tis. (B) GO enrichment analysis of SRGs of pro‐tis. (C) Sankey‐bubble diagram showing the KEGG enrichment analysis SRGs in pro‐org. (D) Sankey‐bubble diagram showing the GO enrichment analysis SRGs in pro‐org. (E) Venn diagram displaying 3 common SRGs between pro‐tis and pro‐org (left). The gene expression levels of MUC4, MYLK and PROX1 in endometrium and pro‐org (right) (*n* ≥ 5 biological replicates for each group). Data are presented as mean ± Standard Error of the Mean (SEM) with two‐tailed Student's *t*‐test. Significance: *p < 0.05.

To gain further insights into senescence‐related characteristics in ageing endometrium, we conducted an intersection analysis with cellular senescence‐associated genes derived from the CellAge database [47]. A total of 68 overlapped DEGs were obtained in advanced age pro‐tis, most of which were up‐regulated (Figure S7A, Table S10). Moreover, these 68 genes were taken as a new gene set for further investigation, termed cellular senescence‐associated DEGs (SRGs). As shown in the functional enrichment analysis, these genes were primarily enriched in ageing‐related pathways and terms (e.g. AGE‐RAGE signalling pathway, MAPK signalling pathway, cellular senescence, regulation of cell death and regulation of cell population proliferation) as well as inflammation‐related pathways (e.g. IL17 signalling pathway and TNF signalling pathway). This suggests that the senescence phenotype of endometrium is strongly associated with cell cycle regulation and inflammation (Figure 3A,B).

In the same way, pro‐org and sec‐org showed similar senescence‐related transcriptome signatures as advanced age endometrium (Figures 3C,D and S7B–D, Tables S10 and S11). The intricate mapping relationship between genes and pathways displayed in the Sankey diagram confirmed that MAPK12 and AKT3 corresponded to the most KEGG pathways, indicating a crucial role in communicating the complicated cross‐talk between different pathways (Figure 3C). In addition, three overlapping genes, including MUC4, MYLK and PROX1, were further obtained by intersecting the SRGs in pro‐tis with SRGs in pro‐org, which were noticeably up‐regulated in both endometrium and pro‐org of advanced age (Figure 3E). All these three genes were pro‐fibrotic, suggesting a strong correlation between endometrial fibrosis and age.

In order to verify the senescence phenotype in advanced age endometrium, we detected the expression of SA‐β‐gal, P53 and Ki67 in endometrium, in which the expression levels of SA‐β‐gal and p53 were apparently increased while the expression level of Ki67 decreased in the advanced age group, confirming the specific role of cell cycle arrest in endometrial ageing (Figure S8).

### 3.4 PI3K‐AKT‐FOXO1 Signalling Pathway Was Activated in Advanced Age Endometrium

**FIGURE 4:**
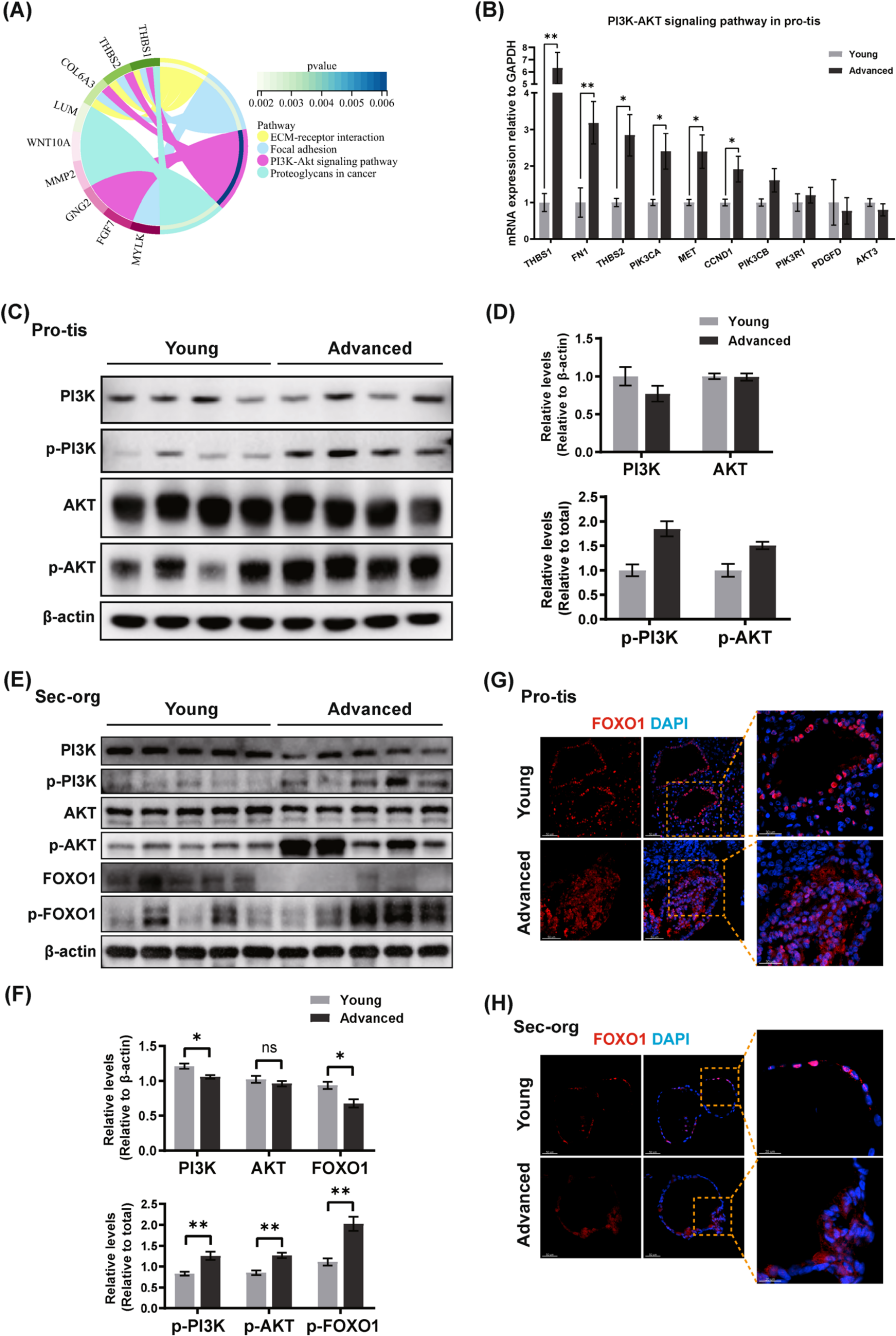
PI3K‐AKT‐FOXO1 signalling pathway was activated in advanced age endometrium. (A) KEGG circle plot showing the pathways enriched by 53 genes screened from up‐regulated DEGs in pro‐tis, pro‐org and sec‐org of advanced age group. (B) The mRNA expression levels of key genes in PI3K‐AKT signalling pathway in pro‐tis (*n* ≥ 6 biological replicates for each group). (C) Representative WB images of PI3K, p‐PI3K, AKT, p‐AKT in pro‐tis. (D) Relative levels of PI3K, p‐PI3K, AKT, p‐AKT, FOXO1, p‐FOXO1 in pro‐tis (*n* = 4 biological replicates for each group). (E) Representative WB images and relative levels of PI3K, p‐PI3K, AKT, p‐AKT, FOXO1, p‐FOXO1 in sec‐org. (F) Relative levels of PI3K, p‐PI3K, AKT, p‐AKT, FOXO1, p‐FOXO1 in sec‐org (*n* = 5 biological replicates for each group). (G) Fluorescence images of FOXO1 in pro‐tis. Scale bar: Left 50 μm, right 50 μm. (H) Fluorescence images of FOXO1 in sec‐org. Scale bar: Left 50 μm, right 50 μm. Data are presented as mean ± standard error of the mean (SEM) with two‐tailed Student's *t*‐test in (B–D). *p < 0.05, **p < 0.01.

To gain deeper insights into the potential mechanisms underlying the impact of ageing on the endometrium, our focus turned towards an up‐regulated signalling pathway: PI3K‐AKT‐FOXO1 pathway. The PI3K‐AKT pathway is a key signalling cascade that regulates diverse cellular functions related to cell proliferation, differentiation, metabolism, longevity and cellular senescence [48]. Previous studies have highlighted that PI3K‐AKT signalling pathway is associated with the invasion of trophoblasts, placental angiogenesis, epithelial mesenchymal transition and decidualization [49, 50, 51]. As expected, KEGG enrichment analysis of 53 up‐regulated common genes among advanced age pro‐tis, pro‐org and sec‐org showed activation of the PI3K‐AKT signalling pathway, suggesting that PI3K‐AKT pathway may play a vital role in endometrial ageing (Figure 4A, Table S6). Subsequent qRT‐PCR confirmed increased transcript levels of key genes in the PI3K‐AKT signalling pathway in endometrium and organoids of advanced age (Figures 4B and S9A,B). Furthermore, we observed an increased level of phosphorylation of PI3K and AKT in the pro‐tis and sec‐org of advanced age group (Figure 4C–F). This suggests that changes in this signalling pathway, unlike imbalanced inflammatory status, are not dependent on the menstrual cycle.

Numerous evidence showed that focal adhesion kinase (FAK, encoded by *PTK2*) serves as a mechanical connection to the ECM and acts as a biochemical signal hub to direct numerous signal proteins such as PI3K/AKT signalling and plays an important role in many biological processes, including fibrosis, inflammation and tumour metastasis [28, 52, 53]. Based on the previous studies and findings, we speculated that accumulated ECM components of advanced age endometrium activate FAK, which phosphorylated downstream PI3K/AKT signalling. In order to validate our speculation, we detected PTK2 mRNA expression and found no difference between the two groups, consistent with our transcriptome data (Figure S9C). However, we observed increased levels of phosphorylation of FAK in the pro‐tis and sec‐org of advanced age group (Figure S9D–F). This suggested that FAK was activated in advanced age endometrium.

FOXO1, as a potential suppressors of fibrosis and a key regulatory transcription factor for receptivity, can be phosphorylated directly by AKT. This modification leads to translocation of FOXO1 from nucleus to cytoplasm, where its activity is inhibited by proteasome‐induced degradation, thus reducing transcriptional activity [54, 55, 56, 57]. We observed reduced expression and elevated phosphorylation levels, as well as significant cytoplasmic translocation of FOXO1 in both advanced age endometrium and organoids, which may lead to exacerbating fibrosis and abnormal receptivity (Figure 4E–H). Therefore, our findings demonstrated that the activation of the PI3K‐AKT signalling pathway phosphorylates FOXO1, resulting in cytoplasmic translocation and inactivation, which could give rise to fibrosis and a decline of endometrial receptivity.

### 3.5 PI3K‐AKT Inhibition Rescues Endometrial Dysfunction While Its Activation Accelerates Aging in Endometrial Organoids

**FIGURE 5:**
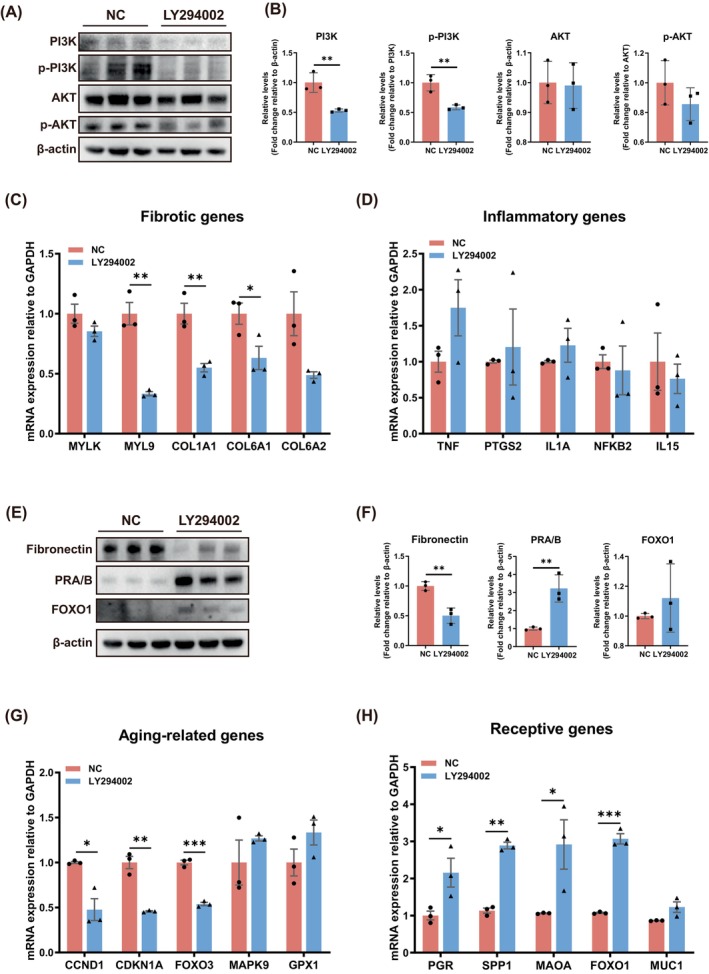
PI3K‐AKT inhibition rescues endometrial dysfunction by attenuating fibrosis, senescence and improving receptivity. (A) Representative WB images of PI3K, p‐PI3K, AKT and p‐AKT in NC and LY294002‐treated organoids. (B) Relative levels of PI3K, p‐PI3K, AKT and p‐AKT in NC and LY294002‐treated organoids. (C) The mRNA expression levels of fibrotic genes in NC and LY294002‐treated organoids. (D) The mRNA expression levels of inflammatory genes in NC and LY294002‐treated organoids. (E) Representative WB images of fibronectin, PRA/B, FOXO1 and p‐FOXO1 in NC and LY294002‐treated organoids. (F) Relative levels of fibronectin, PRA/B and FOXO1 in NC and LY294002‐treated organoids. (G) The mRNA expression levels of ageing‐related genes in NC and LY294002‐treated organoids. (H) The mRNA expression levels of receptive genes in NC and LY294002‐treated organoids. Data are presented as means ± SEM with two‐tailed unpaired Student's *t*‐tests. Significance: *p < 0.05, **p < 0.01, ***p < 0.001.

To investigate the influence of PI3K/AKT signalling on endometrial ageing‐related dysfunction, we treated endometrial organoids derived from three advanced age patients with LY294002 (inhibitor of PI3K/AKT pathway) for 96 h. We observed that the protein levels of p‐PI3K, AKT and p‐AKT in the LY294002‐treated group were remarkedly inhibited, with total AKT expression unchanged (Figure 5A,B). Next, we examined the changes in fibrosis and inflammation of organoids after inhibition of the PI3K‐AKT pathway. According to the findings, blocking the PI3K‐AKT pathway attenuates the mRNA expression of fibrotic genes (*MYL9, COL1A1, COL6A1*) and protein levels of fibronectin (Figure 5C,E,F). However, there was no change observed in the expression of inflammatory genes after LY294002 interference (Figure 5D).

Additionally, organoids exhibited a significant reduction in cell cycle‐related genes (*CDKN1A, CCND1*) after LY294002 treatment, suggesting that inhibition of PI3K‐AKT could partially alleviate cell senescence of endometrium (Figure 5G). Then we induced organoids treated with LY294002 to secretory phase according to the above mentioned. qRT‐PCR assays indicated that the expression of receptive genes (*PGR, SPP1, MAOA, FOXO1*) was rescued by the addition of LY294002(Figure 5H). Similar trends were observed in WB assays (Figure 5E,F). The mentioned results indicated that the PI3K‐AKT pathway is involved in regulating endometrial ageing.

Next, we treated endometrial organoids with 740Y‐P (a specific PI3K activator) and SC‐79 (a specific AKT activator). It was observed through qRT‐PCR that the expression of fibrotic genes (*LAMC3, COL6A1*) and inflammatory genes (*NFKB2, IL1B, PTGS2*) slightly increased with the addition of 740Y‐P and SC79 (Figure S10A,B). Based on these findings, we demonstrated that PI3K‐AKT activation increases fibrosis and inflammation burden in endometrial organoids.

Furthermore, PI3K‐AKT pathway activators‐treated organoids exhibited an ageing phenotype as evidenced by increase expression of ageing‐related genes and protein (Figure S10C,D). We simultaneously induced organoids treated with PI3K‐AKT activators to secretory phase and took note of the fact that the expression of most receptive genes reduced by the addition of 740Y‐P or SC79 (Figure S10E). Immunofluorescence further confirmed significant cytoplasmic translocation of FOXO1 in activators treated organoids (Figure S10F). On the evidence of the above data, it can be inferred that PI3K‐AKT signalling pathway is involved in ageing‐related endometrial dysfunction via FOXO1.

**FIGURE 6:**
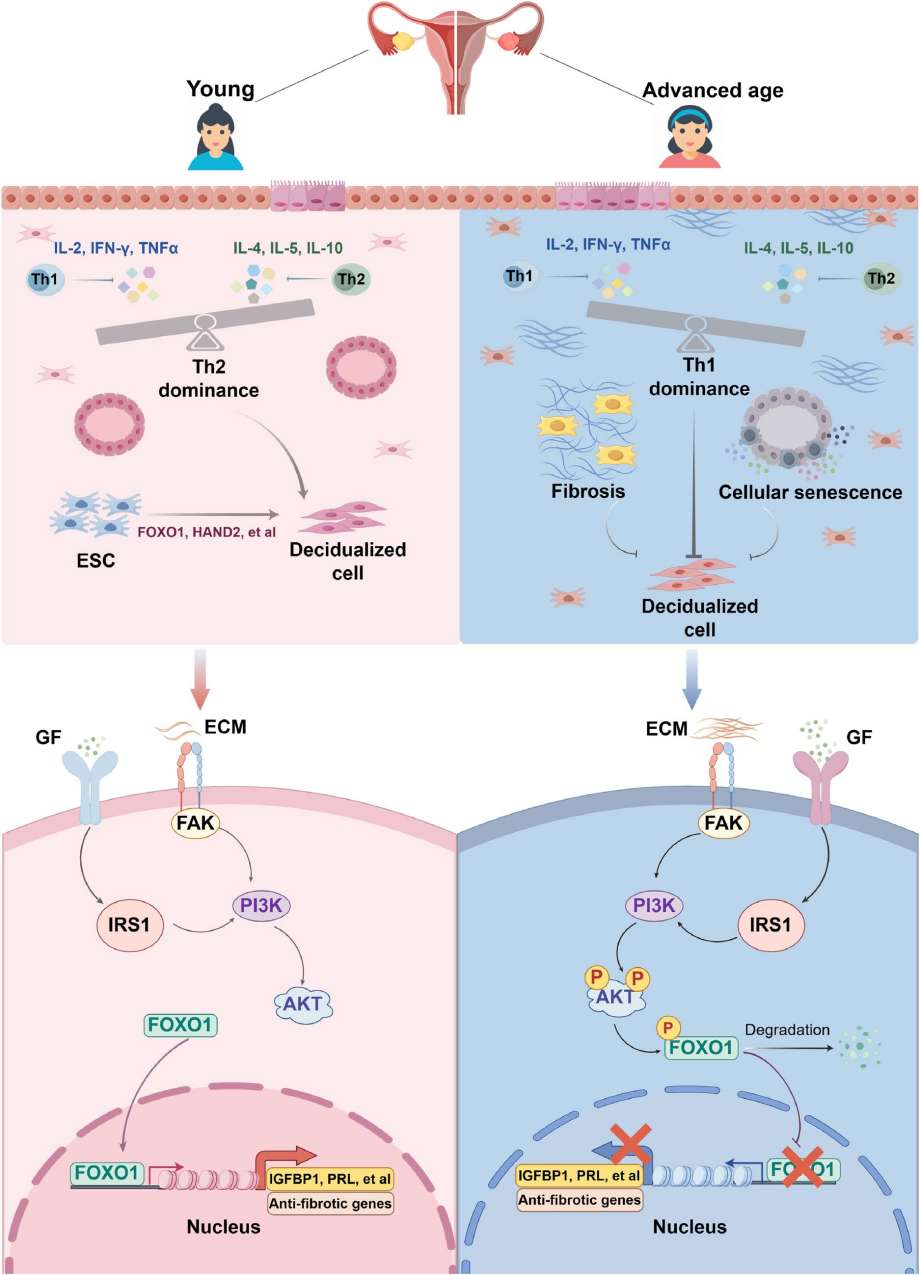
Schematic diagram displaying and summarising the effects of ageing on the endometrium. Image was created by Figdraw.

In summary, our findings collectively showcase that age‐associated decline of endometrial function including fibrosis, inflammatory imbalance and defective endometrial receptivity has already presented in reproductive age. This may potentially impact embryo implantation. To unravel the mechanism of endometrial ageing, we found that the PI3K‐AKT‐FOXO1 signalling pathway was activated in advanced age endometrium, which could give rise to fibrosis and impair endometrial receptivity (Figure 6). Inhibition of this pathway with LY294002 rescued ageing‐related dysfunction of endometrial organoid, whereas the activation of this pathway using 740Y‐P and SC79 accelerates ageing and impairs endometrial receptivity.

## 4 Discussion

In the present study, we focused on functional and signalling pathway alterations and potential molecular mechanisms during endometrial ageing through transcriptome analysis and experiment verification. In this study, we used 35 years as the cutoff to divide young and advanced age groups for the following considerations. First, Devesa‐Peiro et al. utilised artificial intelligence methods to define age groups based on endometrial transcriptomic data and revealed that age‐related changes in gene expression occurred abruptly at 35 years old [17]. Besides, several studies performed in oocyte donation IVF cycles demonstrated women aged 35–42 years old experience a decrease in both implantation rate and live birth rate compared to women under 35, highlighting the impact of advanced age on reproductive outcomes [58, 59]. Therefore, we are more interested in knowing whether age‐related differences of endometrium microenvironment and function already existed in reproductive age, with a view to exploring the impact of endometrial ageing on reproductive health.

The most apparent morphological characteristic in the ageing endometrium is the deposition of collagen with associated tissue fibrosis, which has been reported to be associated with reproductive dysfunction [25, 60, 61, 62]. The association between collagen deposition and inflammation is thought to be an important reason for fibrosis [63, 64], we did notice a significant up‐regulation of pro‐inflammatory pathways in advanced age endometrium and pro‐org, which may be a contributory factor to the development of fibrosis. In addition, a prior study has demonstrated that elevated levels of inflammatory cytokines, acting downstream of NF‐kappa B, including TNFα, IL1β and IL2, play a key role in ovarian ageing [65]. This suggests that there may be a potential link between these cytokines and endometrial ageing.

However, we found that production of pro‐inflammatory cytokines decreased in advanced age sec‐org, accompanied by a Th1 bias. Cytokines play an important role in the process of implantation. Th1 cells mainly secrete IL‐2, IFNγ and TNF‐α, while Th2 cells mainly produce IL‐4, IL‐5 and IL‐10 [66]. It is widely accepted that pregnancy is characterised by a Th2‐dominant cytokine pattern, whereas Th1 immune responses are significantly increased in women with recurrent pregnancy loss [41, 43, 45]. From our aforementioned experiment results and analysis, we confirmed that there is an imbalance of inflammatory homeostasis, as well as an accumulation of chronic inflammation in the ageing endometrium.

In exploring the mechanism of endometrial ageing, we found that PI3K‐AKT signalling pathway was activated in ageing endometrium. The PI3K‐AKT signalling pathway is involved in a variety of cell communications. Recent studies have found that the PI3K‐AKT pathway is associated with the functional regulation of pregnancy‐related factors such as migration of trophoblasts, epithelial mesenchymal transition and endometrial receptivity [49]. Down‐regulation of PI3K‐AKT signalling is required for decidualization [50, 51]. Furthermore, conformational changes in the focal adhesions activate the intracellular kinase FAK, which have been implicated in PI3K‐AKT activation [28, 52, 53]. Guided by these insights, we proposed the hypothesis that the overactivation of FAK‐PI3K‐AKT pathway in aged endometrium may impair decidualization and performed experimental validation.

FOXO1 is a transcription factor that has been shown to play an important role in decidualization [56, 57, 67]. It was shown that phosphorylation of FOXO1 at Ser256 residue makes it a target for ubiquitination‐mediated protein degradation, and AKT is involved in the phosphorylation of FOXO1 at this site [55]. Our results confirmed elevated phosphorylation levels and significant cytoplasmic translocation of FOXO1 in aged endometrium. Additionally, there is growing evidence revealing that FOXO1 exerts an inhibitory effect on fibroblast activation, thereby alleviating the level of fibrosis in many organs, including the liver and kidney [39, 51, 52]. Through the above analysis, we conjectured that overactivation of PI3K‐AKT‐FOXO1 signalling pathway in aged endometrium contributes to FOXO1 degradation, thus impairing decidualization and aggravating fibrosis, consequently in turn adversely impacts endometrial receptivity.

Over the past decades, several therapeutic interventions targeting endometrial ageing have been explored [20, 68, 69]. Nevertheless, therapeutic strategies to combat endometrial ageing are relatively limited due to elusive cellular and molecular signalling mechanisms of endometrial ageing involved. Besides, these agents need to be validated in research models that are closer to real physiological environments such as organoids. We have demonstrated that endometrial organoids can mimic endometrial ageing process in vitro, which can enable us to explore the mechanisms of ageing in depth and screen for potential therapies.

However, this study also has some limitations that must be acknowledged. First, this study is limited in size, although we utilised many molecular experiments to enhances reproducibility of our results. Second, due to the high heterogeneity of clinical samples and limited types of cytokines that kits can detect, we need to further substantiate the inflammatory cytokine profile across menstrual cycle in ageing endometrium through secretome analysis. Besides, given the pivotal role of types and proportions of immunocytes in the endometrium, single‐cell sequencing is still necessary to obtain the landscape of major cell types and intercellular communication atlas. Nonetheless, our findings that a menstrual cycle‐dependent imbalanced inflammatory status exists in ageing endometrium still shed innovative light on the mechanism of an abnormal immune microenvironment in ageing endometrium. Finally, we noticed overactivation of the PI3K‐AKT‐FOXO1 signalling pathway in ageing endometrium, but further studies are needed to investigate the direct effects of this signalling pathway on endometrial function and discover potential intervention targets.

## 5 Conclusion

Taken together, our study unravels that age‐associated decline of endometrial function including fibrosis and diminished receptivity is already present in reproductive age, in line with mounting evidence from other studies. Changes in molecular processes affected by age, including dysregulated inflammatory balance, cellular senescence and abnormal signalling in key pathways, are observed and validated by experiment using endometrium tissue and organoids. To our knowledge, this work is the first to identify the involvement of the PI3K‐AKT‐FOXO1 signalling pathway in ageing endometrium and its close correlation with fibrosis and impaired receptivity characteristics of ageing endometrium. These findings provide new insights into the mechanisms underlying endometrial ageing and provide potential targets for intervention.

## Author Contributions

Minghui Lu, Yu Zhang and Rusong Zhao designed the study. Minghui Lu performed the majority of experiments. Yanli Han helped with the transcriptome analysis. Yining Su, Ruijie Yu and Xueyao Chen helped with data analysis. Tao Li provided human endometrium samples. Minghui Lu wrote the manuscript. Rusong Zhao, Boyang Liu, Tao Li and Han Zhao performed critical revisions. All authors discussed and revised on the manuscript.

## Conflicts of Interest

The authors declare no conflicts of interest.

## Supporting information


**Figure S1.** Establishment and validation of endometrial organoids. (A) Representative images of organoids cultured in vitro. Scale bar = 200 μm. (B) Representative fluorescence images for E‐cad, vimentin, FOXA2 and Ki67 in endometrium and organoids. Scale bar = 50 μm.

**Figure S2.** Endometrial function is declined in advanced age women. (A) The mRNA expression levels of adhesion molecules in pro‐tis (*n* ≥ 5 biological replicates for each group). (B) Masson staining of the endometrium to reveal pathological changes and levels of fibrosis. (C) The mRNA expression levels of adhesion molecules in pro‐org (*n* ≥ 3 biological replicates for each group). (D) The mRNA expression levels of adhesion molecules in sec‐org (*n* ≥ 5 biological replicates for each group). (E) Representative WB images of fibronectin, MYLK and SMA in pro‐org derived from patients Y1–Y5 and A1–A5. (F) Representative WB images of fibronectin, MYLK in pro‐org derived from patients Y6‐Y10 and A6‐A10. (G) Relative levels of fibronectin, MYLK and SMA in pro‐org derived from young and advanced age patients. (H) Representative WB images of fibronectin, MYLK and SMA in sec‐org derived from patients Y6–Y10 and A6–A10. Scale bar = 50 μm. Data are presented as means ± SEM with two‐tailed unpaired Student’s *t*‐tests. Significance: *p < 0.05, **p < 0.01, ***p < 0.001.

**Figure S3.** Endometrial receptivity is declined in advanced age women. (A) Representative WB images of PRA/B and IGFBP1 in sec‐tis. (B) Relative levels of PRA/B and IGFBP1 in sec‐tis from young and advanced age patients. (C) Representative fluorescence images of PRA/B and IGFBP1 in sec‐org. (D) Mean fluorescence intensities of PRA/B and IGFBP1 per viewfield in sec‐org derived from young and advanced age patients. (*n* = 3 biological replicates for each group). (E) Representative WB images of PRA/B, IHH and IGFBP1 in sec‐org derived from patients Y6–Y10 and A6–A10. Scale bar = 50 μm. Data are presented as means ± SEM with two‐tailed unpaired Student’s *t*‐tests. Significance: *p < 0.05, **p < 0.01, ***p < 0.001.

**Figure S4.** Advanced age endometrium exhibited a fibrosis transcriptome characterisation. (A) Principal component analysis (PCA) plot computed with differentially expressed genes (DEGs) in the bulk transcriptome of pro‐tis (left), pro‐org (middle) and sec‐org (right) belonging to the young and advanced age groups. (B) GSEA of fibrosis‐related GO terms between young and advanced age pro‐tis. (C) GSEA of fibrosis‐related pathways between young and advanced age pro‐tis. (D–G) GSEA of fibrosis‐related pathways between young and advanced age pro‐org. (H) Top 5 GO terms between young and advanced age pro‐org analysed by GSEA.

**Figure S5.** Conjoint analysis of common up‐regulated DEGs from endometrium, pro‐org and sec‐org of advanced age group reveals fibrosis transcriptome characterisation. (A) Venn diagram displaying 122 common DEGs screened from tissue up‐regulated DEGs and pro‐org upregulated DEGs in advanced age group(left). KEGG circle plot showing the pathways enriched by these 122 genes (right). (B) Venn diagram displaying 419 common DEGs screened from pro‐org up‐regulated DEGs and sec‐org upregulated DEGs in advanced age group(left). KEGG circle plot showing the pathways enriched by these 419 genes (right). (C) GO enrichment analysis for genes that were screened from tissue up‐regulated DEGs and pro‐org upregulated DEGs (left), pro‐org up‐regulated DEGs and sec‐org upregulated DEGs (right), respectively.

**Figure S6.** Advanced age endometrium exhibited a menstrual cycle‐specific imbalanced inflammatory status. (A) KEGG enrichment analysis for genes that were up‐regulated in advanced age pro‐org. (B) The mRNA expression levels of inflammatory molecules in pro‐tis (*n* ≥ 6 biological replicates for each group). (C) Representative images of gated flow cytometry scatter plots of target proteins using beads separated by size and dye concentration (up). The quantification of inflammatory cytokines of medium in pro‐org (middle and down, *n* ≥ 4 biological replicates for each group). (D) The quantification of inflammatory cytokines of medium in sec‐org (*n* ≥ 4 biological replicates for each group). (E) The mRNA expression levels of inflammatory molecules in sec‐tis. (F) Representative WB images of NFκBp52, TNFα and c‐JUN in sec‐tis. (G) Relative levels of NFκBp52, TNFα and c‐JUN in sec‐tis from young and advanced age patients. Data are presented as means ± SEM with two‐tailed unpaired Student’s *t*‐tests. *p < 0.05; **p < 0.01; ***p < 0.001.

**Figure S7.** Advanced age endometrium exhibited ageing transcriptome characterisation. (A) Volcano plot displaying the SRGs in pro‐tis. (B) Volcano plot displaying the SRGs in pro‐org. (C) Heatmap presenting relative expression of SRGs in sec‐org. (D) KEGG and GO enrichment analysis of SRGs in sec‐org.

**Figure S8.** The expression levels of cell senescence marker increased in advanced age endometrium. (A) Representative WB images of SA‐β‐gal and p53 in endometrium. (B) Relative levels SA‐β‐gal and p53 in endometrium (*n* = 4 biological replicates for each group). (C) Representative fluorescence images for SA‐β‐gal, p53 and Ki67 in endometrium of young group and advanced age group. (D) Quantification of SA‐β‐gal positive area in endometrium. (*n* = 3 biological replicates for each group). (E) Quantification of p53 positive area in endometrium. (*n* = 3 biological replicates for each group). (F) Quantification of ki67‐positive cells in endometrium. (*n* = 3 biological replicates for each group). Scale bar = 50 μm. A two‐tailed *t*‐test was used for statistical analysis. Data are presented as mean ± standard error of the mean (SEM). *p < 0.05, **p < 0.01, ***p < 0.001.

**Figure S9.** PI3K/AKT/FOXO1 signalling pathway was activated in advanced age endometrium. (A) The mRNA expression levels of key genes in PI3K‐AKT signalling pathway in pro‐org (*n* ≥ 3 biological replicates for each group). (B) The mRNA expression levels of key genes in PI3K‐AKT signalling pathway in sec‐org (*n* ≥ 5 biological replicates for each group). (C) The mRNA expression levels of PTK2 in pro‐tis and sec‐org. (D) Representative WB images of FAK and p‐FAK in pro‐tis from young and advanced age patients. (E) Representative WB images of FAK and p‐FAK in sec‐org derived from young and advanced age patients. (F) Relative levels of FAK and p‐FAK in pro‐tis and sec‐org from young and advanced age patients. Scale bar = 50 μm. A two‐tailed *t*‐test was used for statistical analysis. Data are presented as mean ± standard error of the mean (SEM). *p < 0.05, **p < 0.01, ***p < 0.001.

**Figure S10.** PI3K‐AKT activation accelerates ageing‐related dysfunction in endometrial organoids. (A) The mRNA expression levels of fibrotic genes in NC, 740Y‐P and SC79‐treated organoids. (B) The mRNA expression levels of inflammatory genes in NC, 740Y‐P and SC79‐treated organoids. (C) The mRNA expression levels of ageing‐related genes in NC, 740Y‐P and SC79‐treated organoids. (D) Representative WB images and relative levels of SA‐β‐gal in NC, 740Y‐P and SC79‐treated organoids. (E) The mRNA expression levels of receptive genes in NC, 740Y‐P and SC79‐treated organoids. (F) Representative fluorescence images FOXO1 in NC, 740Y‐P and SC79‐treated organoids. Scale bar = 50 μm (left). Scale bar = 10 μm (right). A two‐tailed *t*‐test was used for statistical analysis. Data are presented as mean ± standard error of the mean (SEM). *p < 0.05, **p < 0.01, ***p < 0.001.

**Table S1.** Clinical information of patients.

**Table S2.** Organoid expansion medium (ExM) composition and hormone treatment details.

**Table S3.** Primer sequences used for quantitative real‐time PCR (qRT‐PCR).

**Table S4.** Antibodies used for immunofluorescence staining and western blotting.

**Table S5.** Differentially expressed genes (DEGs) in the pro‐tis, pro‐org and sec‐org.

**Table S6.** Shared up‐regulated DEGs and enrichment analysis among the pro‐tis, pro‐org and sec‐org.

**Table S7.** GSEA of the fibrosis‐related signalling pathways and inflammation related signalling pathways between the young and advanced age pro‐tis.

**Table S8.** GSEA of the fibrosis‐related signalling pathways and KEGG function enrichment of up‐regulated DEGs in advanced age pro‐org.

**Table S9.** KEGG function enrichment of down‐regulated DEGs in advanced age sec‐org.

**Table S10.** KEGG and GO function enrichment of cellular senescence‐associated DEGs (SRGs) in pro‐tis and pro‐org.

**Table S11.** KEGG and GO function enrichment of SRGs in sec‐org.

## Data Availability

The data that support the findings of this study are openly available in Genome Sequence Archive at https://ngdc.cncb.ac.cn/gsa‐human/s/osvnErF0.
