# Supplementary figures and images for: Investigating Aging‐Related Endometrial Dysfunction Using Endometrial Organoids
Source: Cell Prolif. 2024 Dec 18;58(4):e13780. doi: 10.1111/cpr.13780 (PMC11969247; doi:10.1111/cpr.13780)

Figure S1

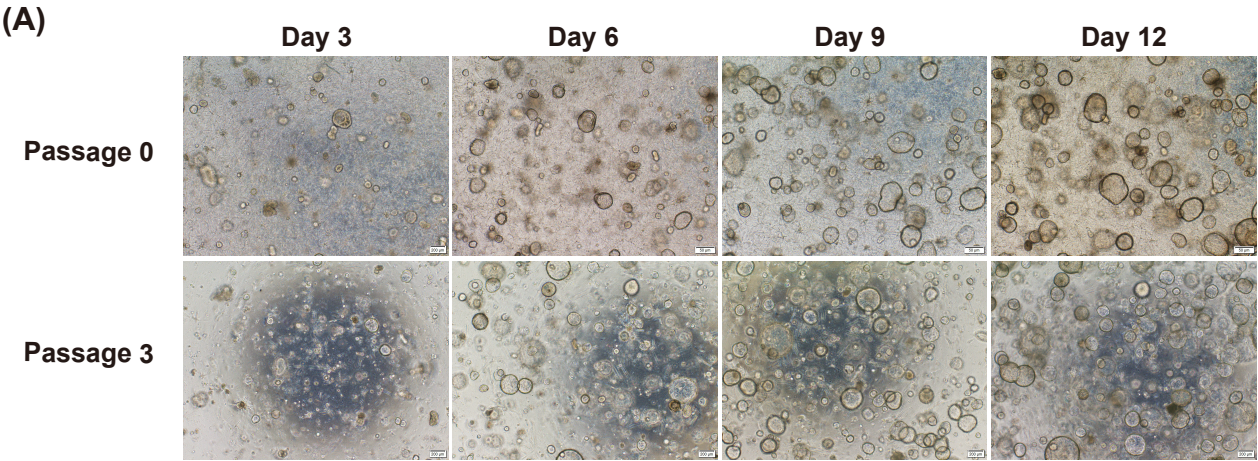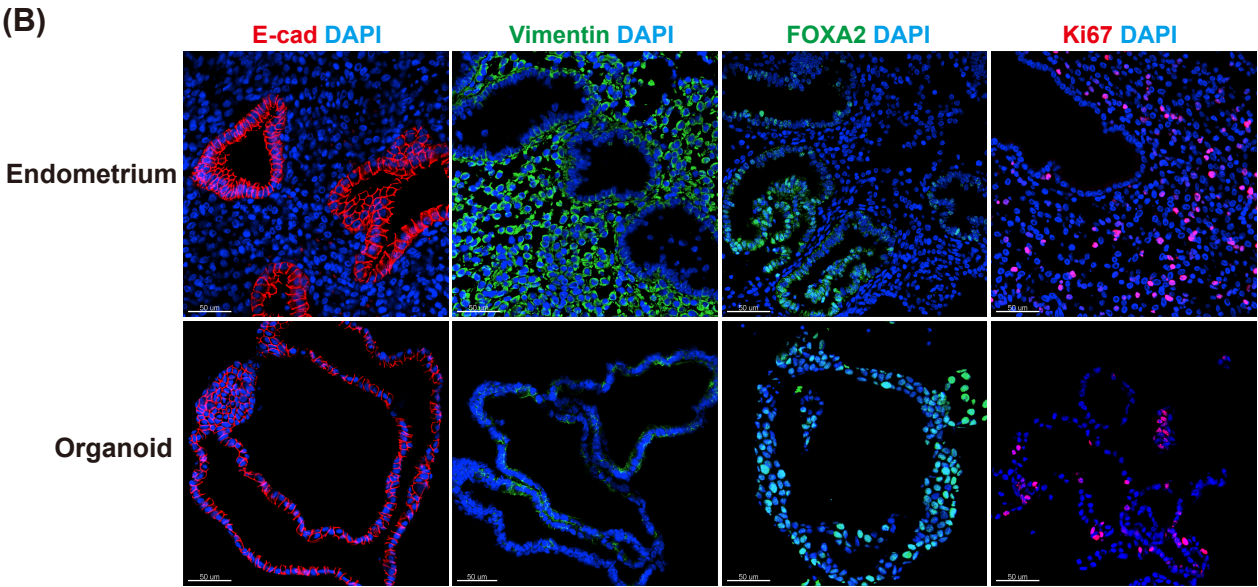

Supplement: Supplementary file 1 — Figure S1. Establishment and validation of endometrial organoids. (A) Representative images of organoids cultured in vitro. Scale bar = 200 μm. (B) Representative fluorescence images for E‐cad, vimentin, FOXA2 and Ki67 in endometrium and organoids. Scale bar = 50 μm. [file CPR-58-e13780-s002.pdf]

Figure S2

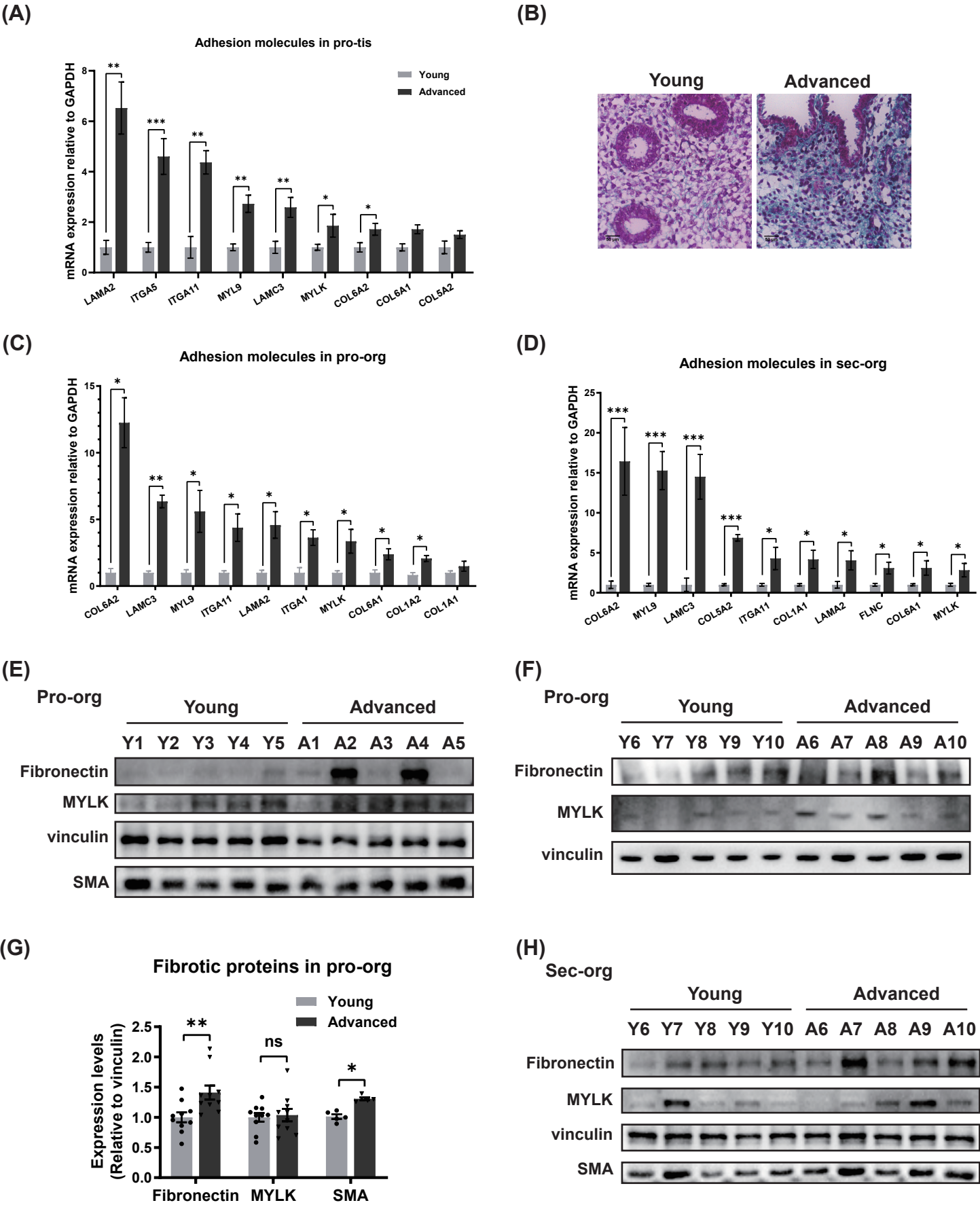

Supplement: Supplementary file 2 — Figure S2. Endometrial function is declined in advanced age women. (A) The mRNA expression levels of adhesion molecules in pro‐tis (n ≥ 5 biological replicates for each group). (B) Masson staining of the endometrium to reveal pathological changes and levels of fibrosis. (C) The mRNA expression levels of adhesion molecules in pro‐org (n ≥ 3 biological replicates for each group). (D) The mRNA expression levels of adhesion molecules in sec‐org (n ≥ 5 biological replicates for each group). (E) Representative WB images of fibronectin, MYLK and SMA in pro‐org derived from patients Y1–Y5 and A1–A5. (F) Representative WB images of fibronectin, MYLK in pro‐org derived from patients Y6‐Y10 and A6‐A10. (G) Relative levels of fibronectin, MYLK and SMA in pro‐org derived from young and advanced age patients. (H) Representative WB images of fibronectin, MYLK and SMA in sec‐org derived from patients Y6–Y10 and A6–A10. Scale bar = 50 μm. Data are presented as means ± SEM with two‐tailed unpaired Student’s t‐tests. Significance: *p < 0.05, **p < 0.01, ***p < 0.001. [file CPR-58-e13780-s001.pdf]

Figure S3

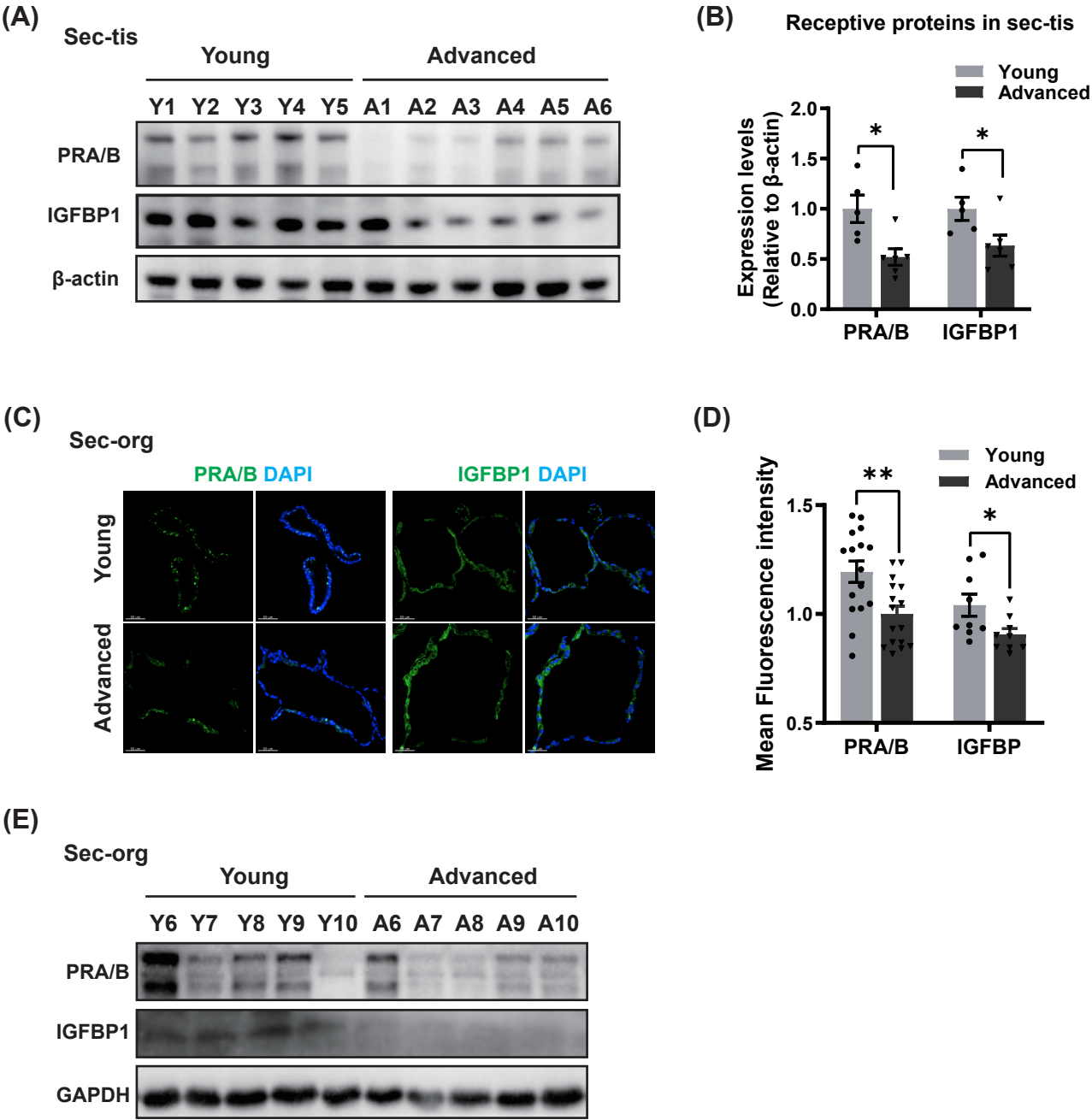

Supplement: Supplementary file 3 — Figure S3. Endometrial receptivity is declined in advanced age women. (A) Representative WB images of PRA/B and IGFBP1 in sec‐tis. (B) Relative levels of PRA/B and IGFBP1 in sec‐tis from young and advanced age patients. (C) Representative fluorescence images of PRA/B and IGFBP1 in sec‐org. (D) Mean fluorescence intensities of PRA/B and IGFBP1 per viewfield in sec‐org derived from young and advanced age patients. (n = 3 biological replicates for each group). (E) Representative WB images of PRA/B, IHH and IGFBP1 in sec‐org derived from patients Y6–Y10 and A6–A10. Scale bar = 50 μm. Data are presented as means ± SEM with two‐tailed unpaired Student’s t‐tests. Significance: *p < 0.05, **p < 0.01, ***p < 0.001. [file CPR-58-e13780-s012.pdf]

Figure S4

(A)

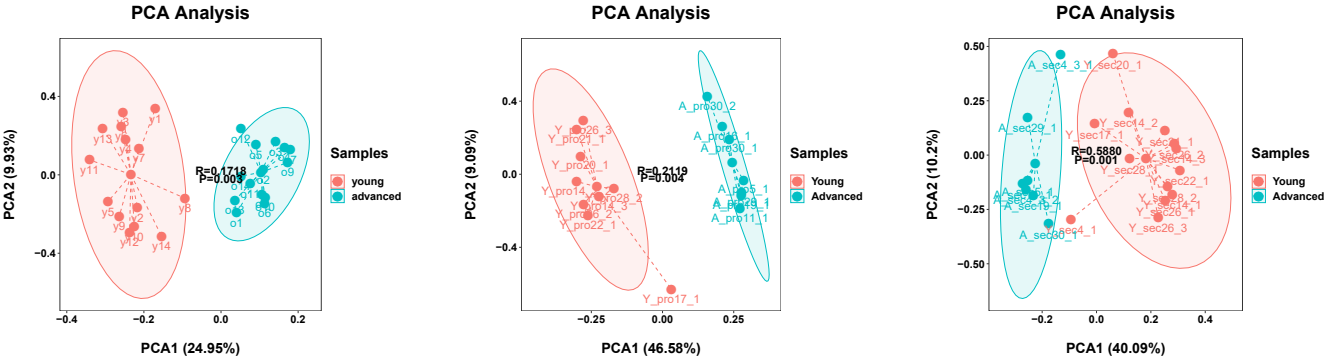

(B)

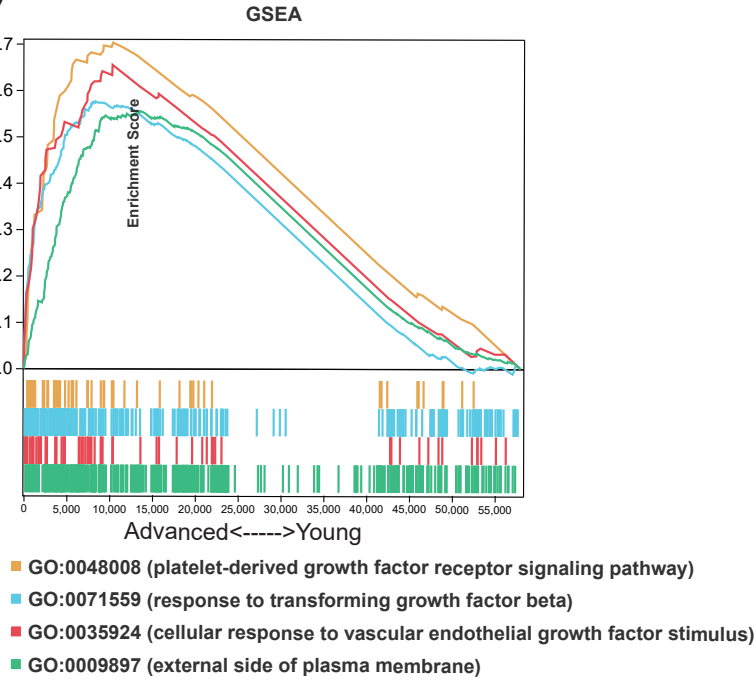

(C)

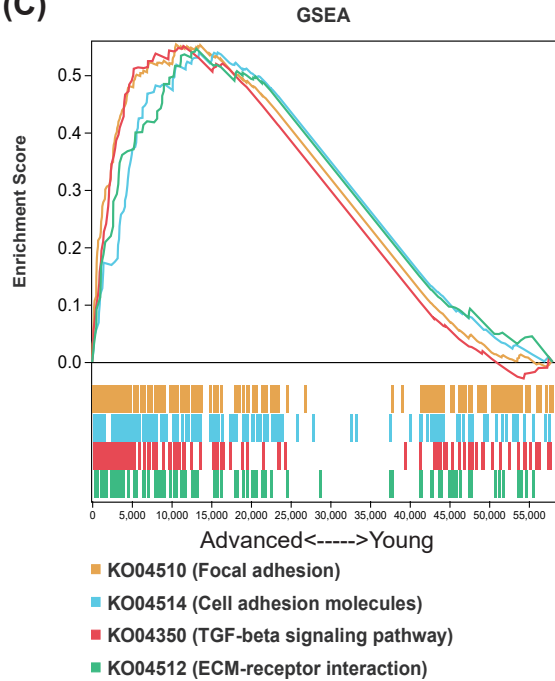

(D)

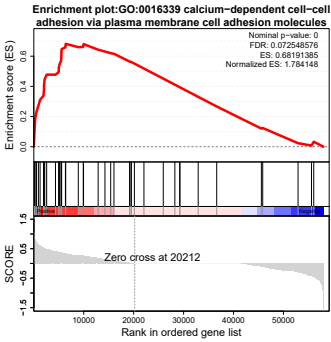

(E)

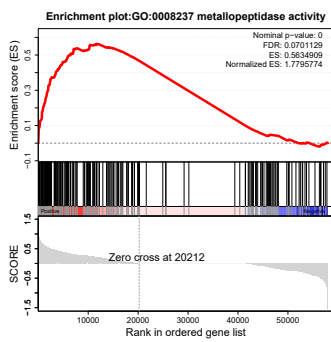

(H)

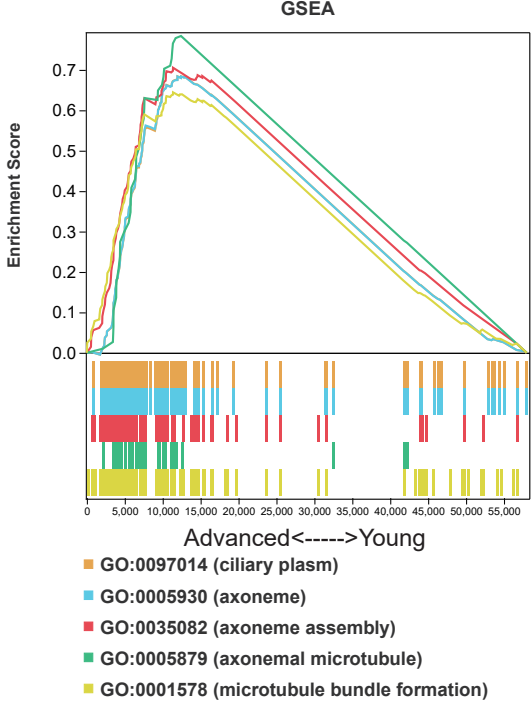

(F)

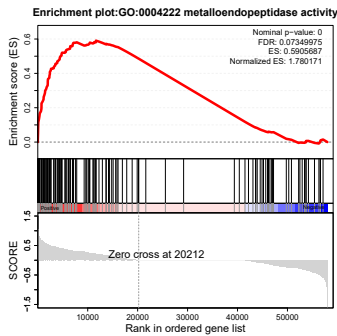

(G)

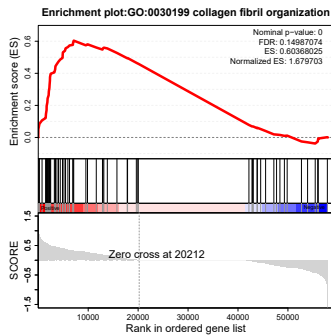

Supplement: Supplementary file 4 — Figure S4. Advanced age endometrium exhibited a fibrosis transcriptome characterisation. (A) Principal component analysis (PCA) plot computed with differentially expressed genes (DEGs) in the bulk transcriptome of pro‐tis (left), pro‐org (middle) and sec‐org (right) belonging to the young and advanced age groups. (B) GSEA of fibrosis‐related GO terms between young and advanced age pro‐tis. (C) GSEA of fibrosis‐related pathways between young and advanced age pro‐tis. (D–G) GSEA of fibrosis‐related pathways between young and advanced age pro‐org. (H) Top 5 GO terms between young and advanced age pro‐org analysed by GSEA. [file CPR-58-e13780-s015.pdf]

Figure S5

(A)

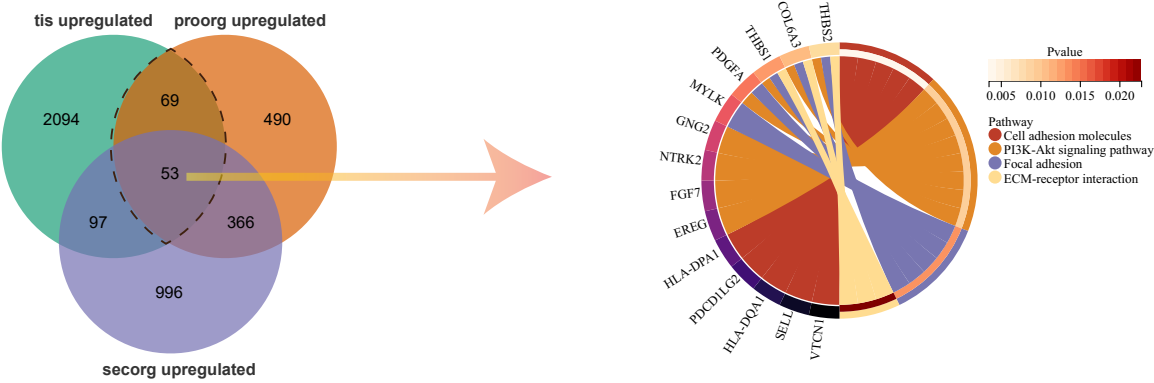

(B)

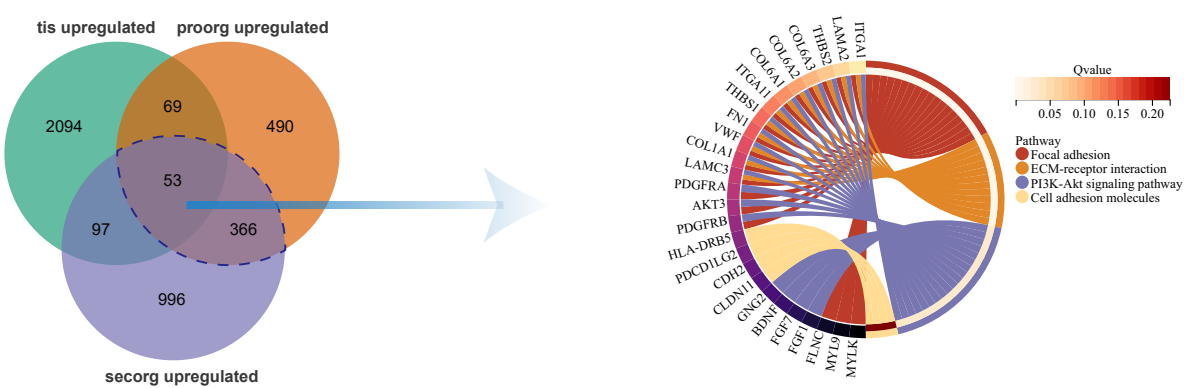

(C)

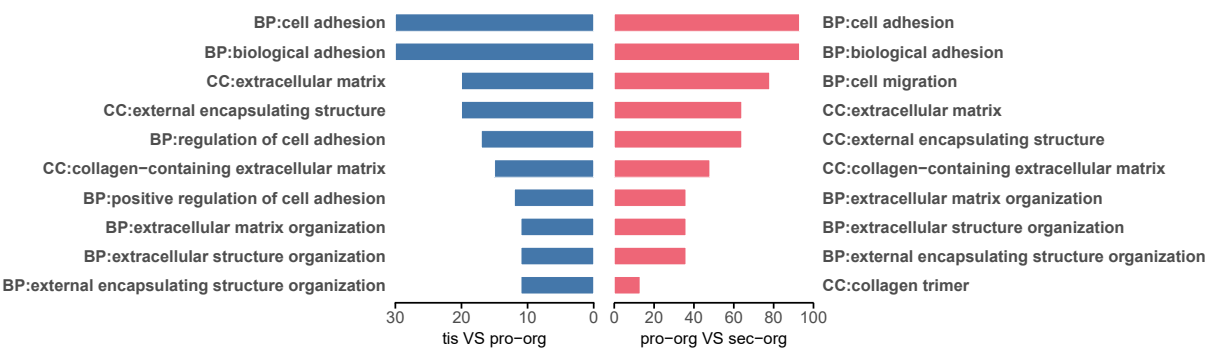

Supplement: Supplementary file 5 — Figure S5. Conjoint analysis of common up‐regulated DEGs from endometrium, pro‐org and sec‐org of advanced age group reveals fibrosis transcriptome characterisation. (A) Venn diagram displaying 122 common DEGs screened from tissue up‐regulated DEGs and pro‐org upregulated DEGs in advanced age group(left). KEGG circle plot showing the pathways enriched by these 122 genes (right). (B) Venn diagram displaying 419 common DEGs screened from pro‐org up‐regulated DEGs and sec‐org upregulated DEGs in advanced age group(left). KEGG circle plot showing the pathways enriched by these 419 genes (right). (C) GO enrichment analysis for genes that were screened from tissue up‐regulated DEGs and pro‐org upregulated DEGs (left), pro‐org up‐regulated DEGs and sec‐org upregulated DEGs (right), respectively. [file CPR-58-e13780-s008.pdf]

**Figure S6**

**(A)**

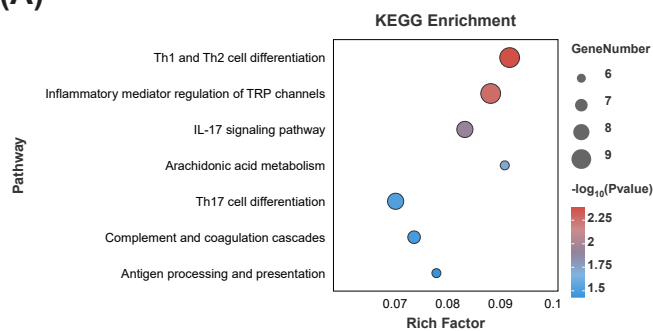

**(B)**

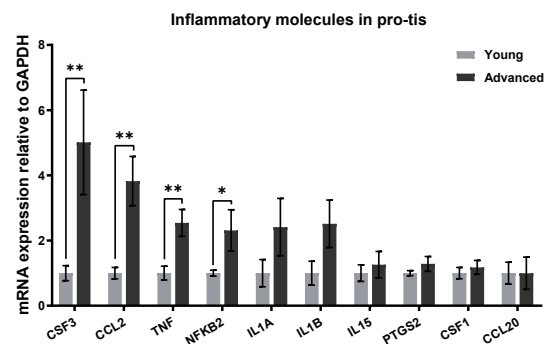

**(C)**

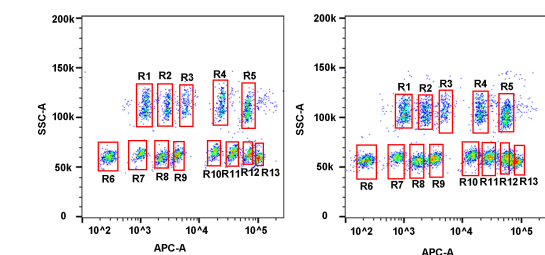

**(D)**

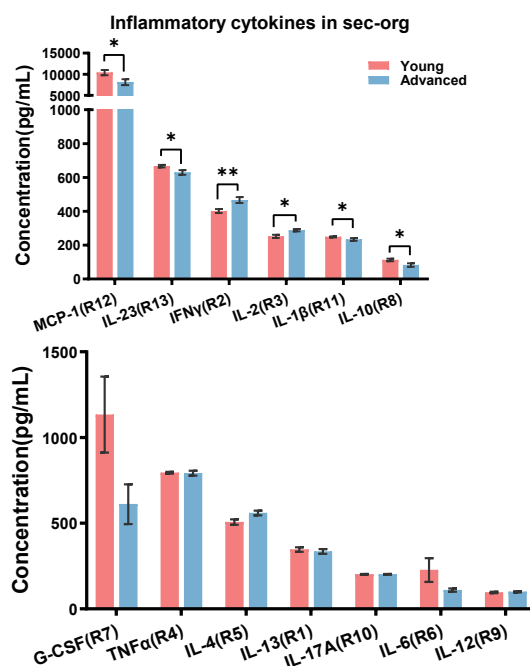

**(E)**

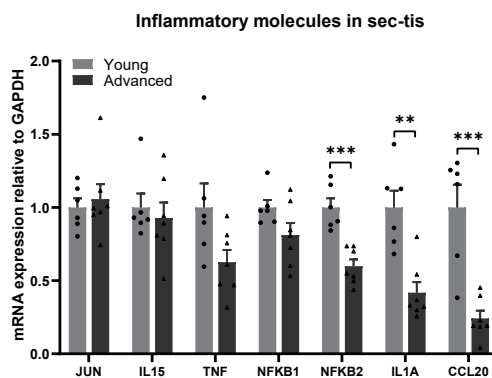

**(F)**

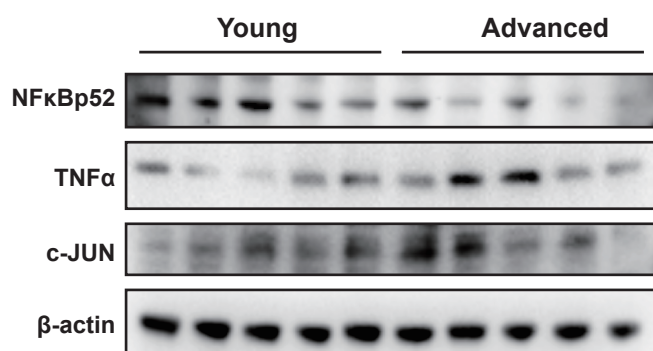

**(G)**

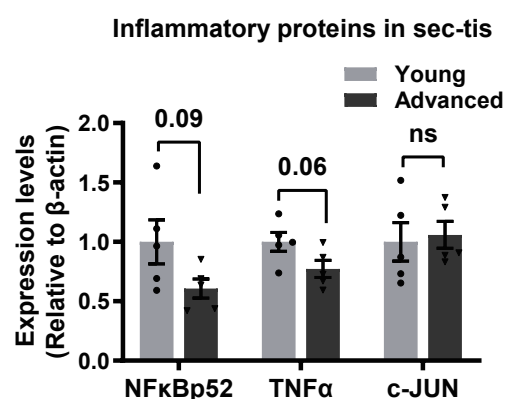

Supplement: Supplementary file 6 — Figure S6. Advanced age endometrium exhibited a menstrual cycle‐specific imbalanced inflammatory status. (A) KEGG enrichment analysis for genes that were up‐regulated in advanced age pro‐org. (B) The mRNA expression levels of inflammatory molecules in pro‐tis (n ≥ 6 biological replicates for each group). (C) Representative images of gated flow cytometry scatter plots of target proteins using beads separated by size and dye concentration (up). The quantification of inflammatory cytokines of medium in pro‐org (middle and down, n ≥ 4 biological replicates for each group). (D) The quantification of inflammatory cytokines of medium in sec‐org (n ≥ 4 biological replicates for each group). (E) The mRNA expression levels of inflammatory molecules in sec‐tis. (F) Representative WB images of NFκBp52, TNFα and c‐JUN in sec‐tis. (G) Relative levels of NFκBp52, TNFα and c‐JUN in sec‐tis from young and advanced age patients. Data are presented as means ± SEM with two‐tailed unpaired Student’s t‐tests. *p < 0.05; **p < 0.01; ***p < 0.001. [file CPR-58-e13780-s005.pdf]

**(A)**

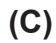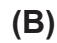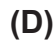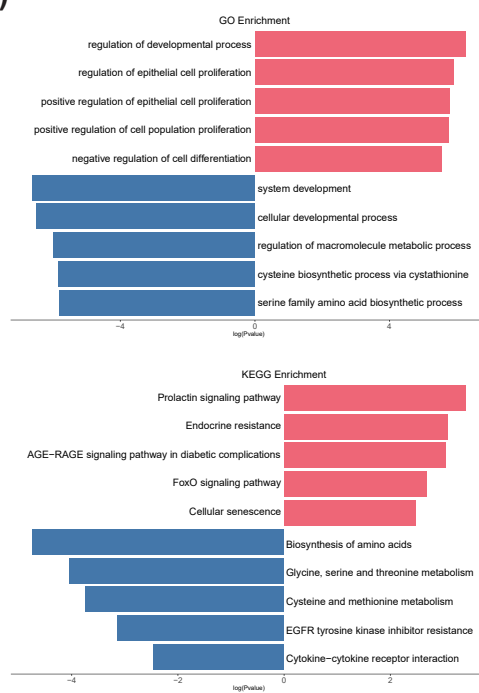

Supplement: Supplementary file 7 — Figure S7. Advanced age endometrium exhibited ageing transcriptome characterisation. (A) Volcano plot displaying the SRGs in pro‐tis. (B) Volcano plot displaying the SRGs in pro‐org. (C) Heatmap presenting relative expression of SRGs in sec‐org. (D) KEGG and GO enrichment analysis of SRGs in sec‐org. [file CPR-58-e13780-s016.pdf]

Figure S8

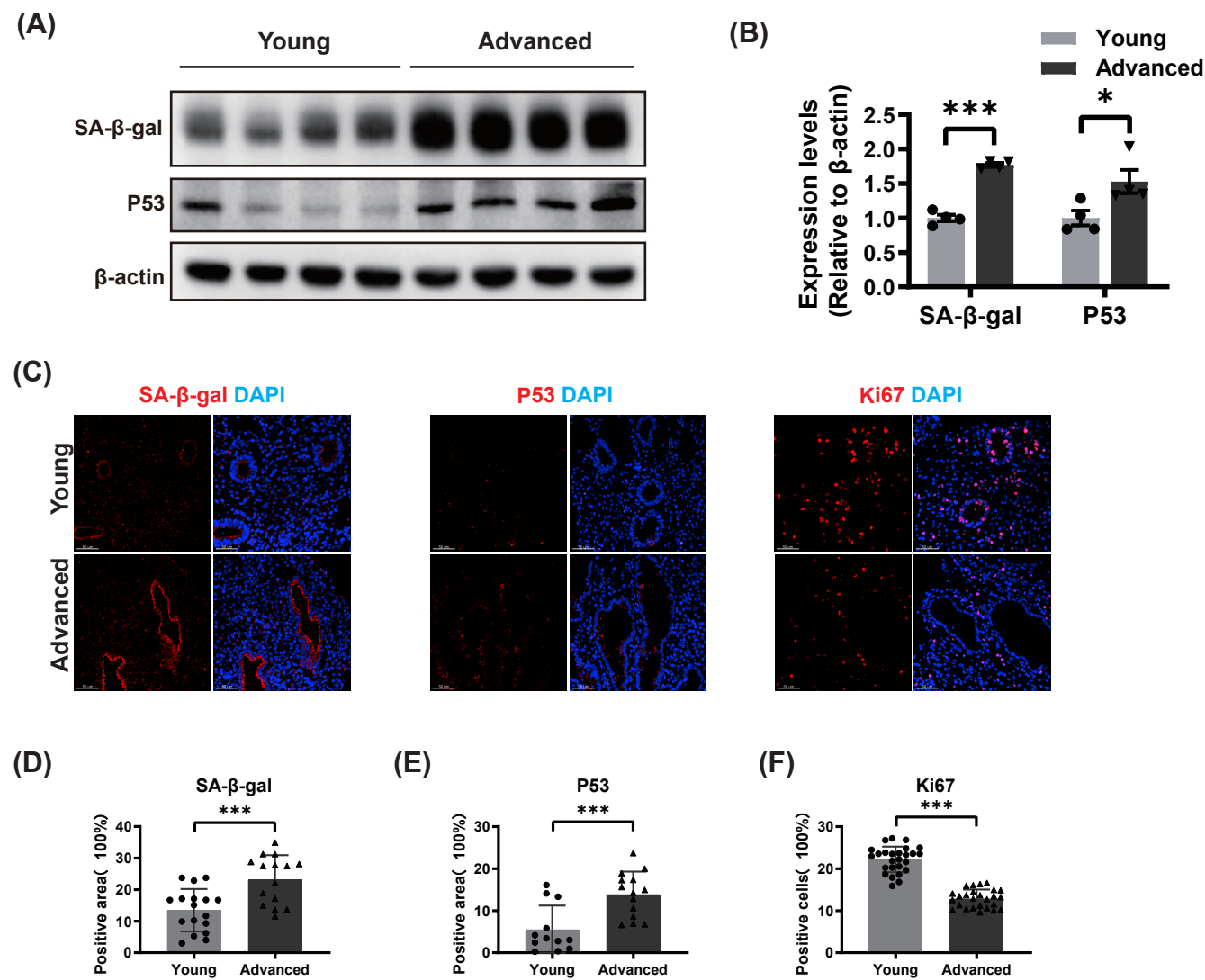

Supplement: Supplementary file 8 — Figure S8. The expression levels of cell senescence marker increased in advanced age endometrium. (A) Representative WB images of SA‐β‐gal and p53 in endometrium. (B) Relative levels SA‐β‐gal and p53 in endometrium (n = 4 biological replicates for each group). (C) Representative fluorescence images for SA‐β‐gal, p53 and Ki67 in endometrium of young group and advanced age group. (D) Quantification of SA‐β‐gal positive area in endometrium. (n = 3 biological replicates for each group). (E) Quantification of p53 positive area in endometrium. (n = 3 biological replicates for each group). (F) Quantification of ki67‐positive cells in endometrium. (n = 3 biological replicates for each group). Scale bar = 50 μm. A two‐tailed t‐test was used for statistical analysis. Data are presented as mean ± standard error of the mean (SEM). *p < 0.05, **p < 0.01, ***p < 0.001. [file CPR-58-e13780-s013.pdf]

**Figure S9**

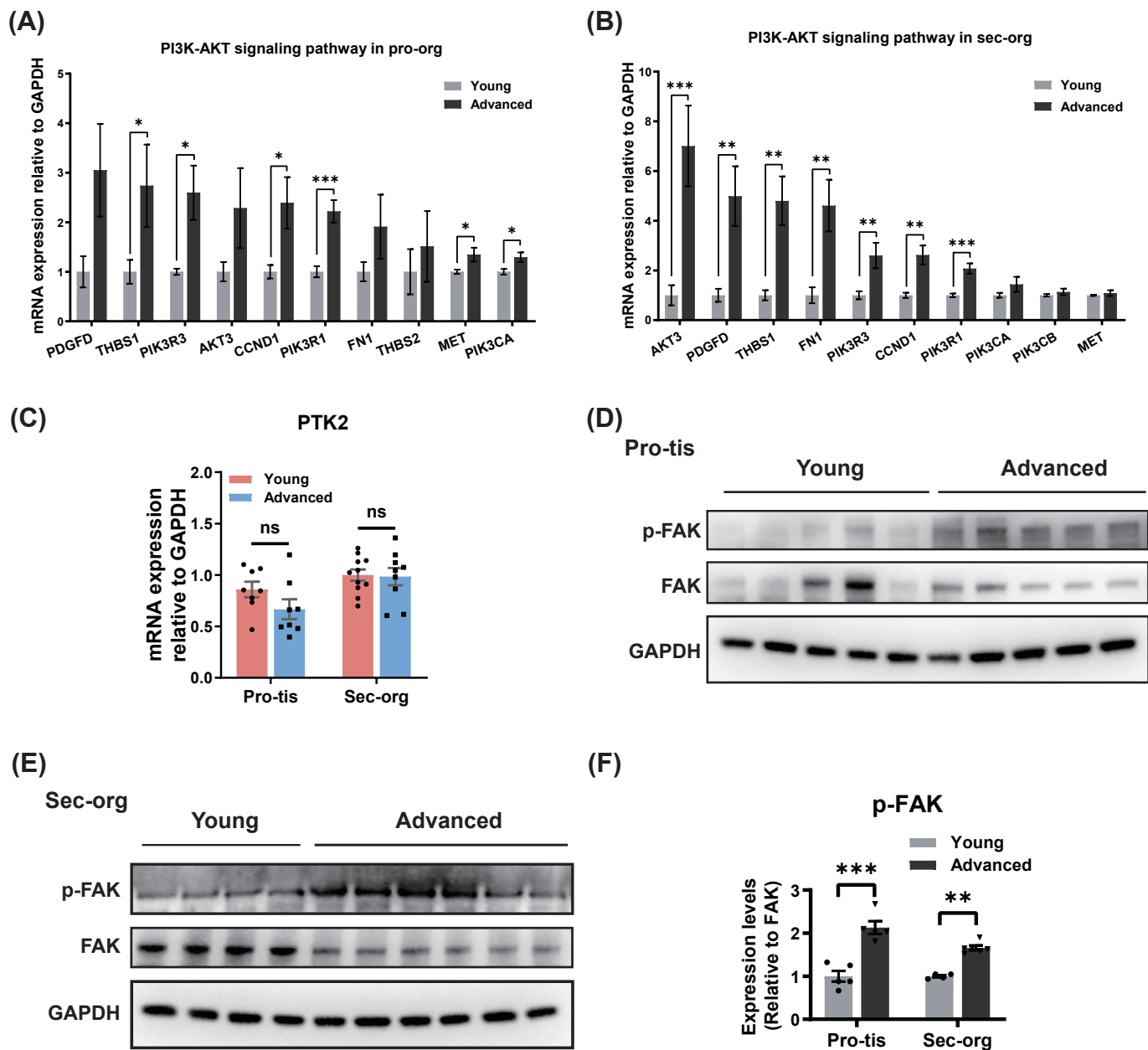

Supplement: Supplementary file 9 — Figure S9. PI3K/AKT/FOXO1 signalling pathway was activated in advanced age endometrium. (A) The mRNA expression levels of key genes in PI3K‐AKT signalling pathway in pro‐org (n ≥ 3 biological replicates for each group). (B) The mRNA expression levels of key genes in PI3K‐AKT signalling pathway in sec‐org (n ≥ 5 biological replicates for each group). (C) The mRNA expression levels of PTK2 in pro‐tis and sec‐org. (D) Representative WB images of FAK and p‐FAK in pro‐tis from young and advanced age patients. (E) Representative WB images of FAK and p‐FAK in sec‐org derived from young and advanced age patients. (F) Relative levels of FAK and p‐FAK in pro‐tis and sec‐org from young and advanced age patients. Scale bar = 50 μm. A two‐tailed t‐test was used for statistical analysis. Data are presented as mean ± standard error of the mean (SEM). *p < 0.05, **p < 0.01, ***p < 0.001. [file CPR-58-e13780-s020.pdf]

Figure S10

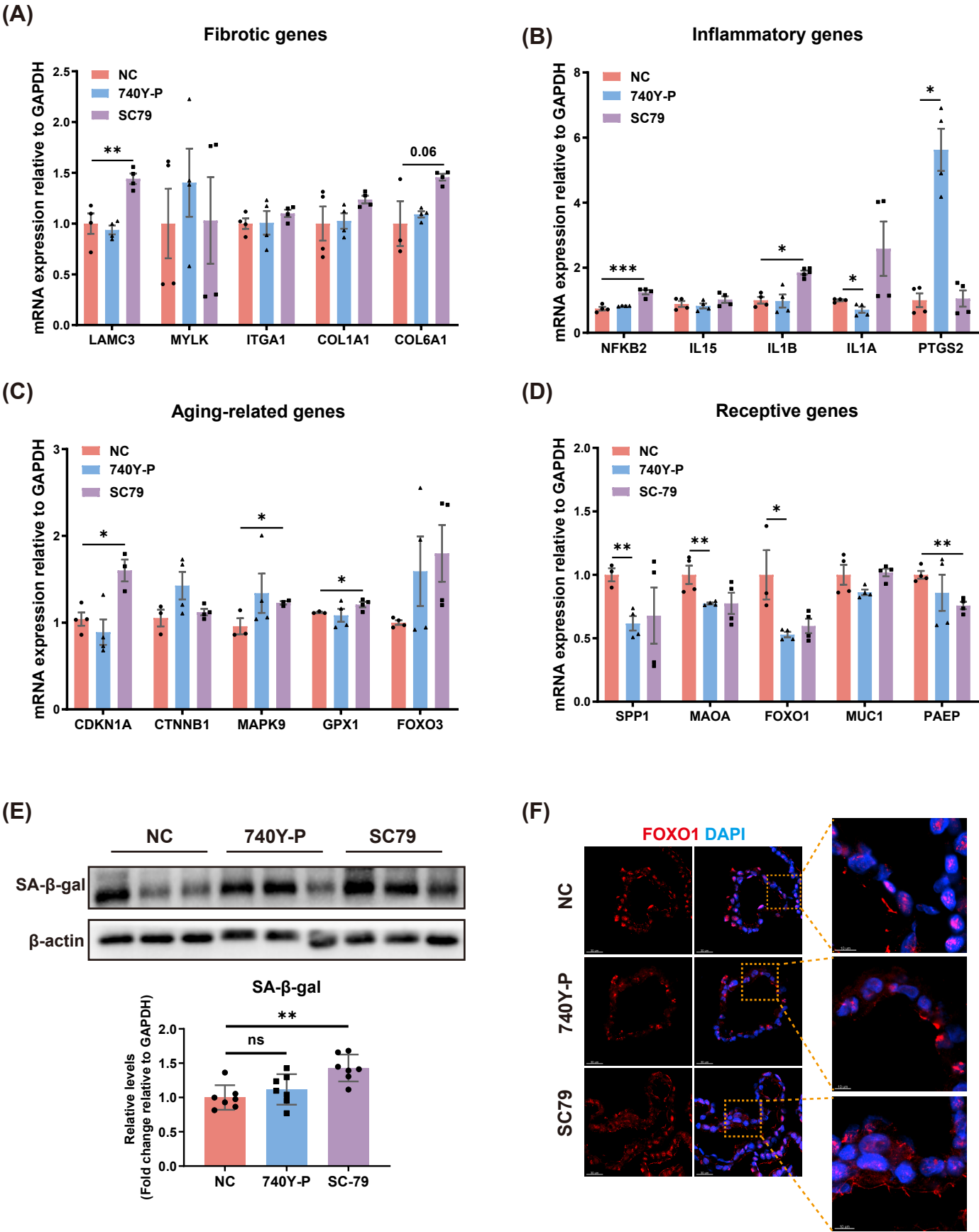

Supplement: Supplementary file 10 — Figure S10. PI3K‐AKT activation accelerates ageing‐related dysfunction in endometrial organoids. (A) The mRNA expression levels of fibrotic genes in NC, 740Y‐P and SC79‐treated organoids. (B) The mRNA expression levels of inflammatory genes in NC, 740Y‐P and SC79‐treated organoids. (C) The mRNA expression levels of ageing‐related genes in NC, 740Y‐P and SC79‐treated organoids. (D) Representative WB images and relative levels of SA‐β‐gal in NC, 740Y‐P and SC79‐treated organoids. (E) The mRNA expression levels of receptive genes in NC, 740Y‐P and SC79‐treated organoids. (F) Representative fluorescence images FOXO1 in NC, 740Y‐P and SC79‐treated organoids. Scale bar = 50 μm (left). Scale bar = 10 μm (right). A two‐tailed t‐test was used for statistical analysis. Data are presented as mean ± standard error of the mean (SEM). *p < 0.05, **p < 0.01, ***p < 0.001. [file CPR-58-e13780-s004.pdf]
